# Network rewiring conserves the topology of drought-impaired food webs

**DOI:** 10.1038/s42003-025-09035-2

**Published:** 2025-11-24

**Authors:** Athen Ma, Pavel Kratina, Mark E. Ledger, Eoin J. O’Gorman

**Affiliations:** 1 School of Electronic Engineering and Computer Science, Queen Mary University of London, London, UK; 2 School of Biological and Behavioural Sciences, Queen Mary University of London, London, UK; 3 School of Geography, Earth and Environmental Sciences, University of Birmingham, Edgbaston, Birmingham, UK; 4 School of Life Sciences, University of Essex, Wivenhoe Park, Colchester, UK

**Keywords:** Food webs, Ecological networks, Freshwater ecology

## Abstract

Extreme climatic events such as drought are increasing in magnitude and frequency, representing one of the biggest threats to freshwaters across the globe. Although drought can cause extensive loss or turnover of biodiversity, food web structure often remains surprisingly unchanged. This topological constancy suggests that ecosystems undergo rewiring of biotic interactions resulting from adaptive species responses, although how compensatory mechanics collectively reorganise food webs are largely unknown. Here, we perform a merging of trophic ecology with an approach from network science (global network alignment, which optimises network comparison and reveals restructuring) to assess the impact of experimental drought on the topology of stream food webs. We found that whilst drought caused substantial biodiversity loss, trophic plasticity among the surviving consumers conserved 80% of the original food web topology, maintaining connectance and in turn stability. This structural inertia was driven by extensive rewiring among the surviving species, but in contrast to expectations, we observed considerable trophic plasticity among dietary specialists who in fact disproportionally rewired more than their generalist counterparts. These findings demonstrate that adaptive dietary shifts among specialist species play an underappreciated role in mitigating the effects of drought and governing the topological persistence of ecological networks.

## Abstract (web summary)

Network alignment of food webs reveals systemwide adaptive dietary shift as a key mechanism for species to persist biodiversity loss and physiological stress under drought, with specialist species proportionally expanded their diets the most.

## Introduction

Severe temperatures have been recorded across the globe in recent years which has led to extreme drought in many countries, serving a stark reminder of the growing threat posed by climate change. These extreme climatic events can have profound effects on both the composition of species and the interactions among them^1,2^, such as biodiversity loss and compensatory re-assembly as more adaptive species switch and expand their diets^3^, triggering changes in the structural organisation and energy fluxes in food webs^4^. For example, reduced complexity^5^, shorter food chain^6^ and less efficient energy fluxes^7^ have been previously reported following an environmental stressor.

A common way to assess how ecosystems respond to perturbations like drought is to measure changes in the structural organisation of food webs^8,9^. Network analysis has been used increasingly to examine the topology of food webs^9–11^, as many metrics offer insights into how the complexity, functioning, and dynamics of ecosystems respond to environmental change. For example, connectance has been widely used as a proxy for food web complexity because it captures the density of species interactions^9^, and it has been found to vary along environmental gradients^5^. Such findings are not universal, however, with topological constancy reported in many studies despite significant species turnover in response to environmental stressors^12–14^, providing evidence of topological conservation through re-organisation of trophic links. Hence, a better understanding of how species undergo dietary shifts and how these compensatory mechanics among species collectively rebalance structural organisation in ecological networks will greatly enhance our ability to assess and predict ecosystem responses to environmental change.

**Fig. 1:**
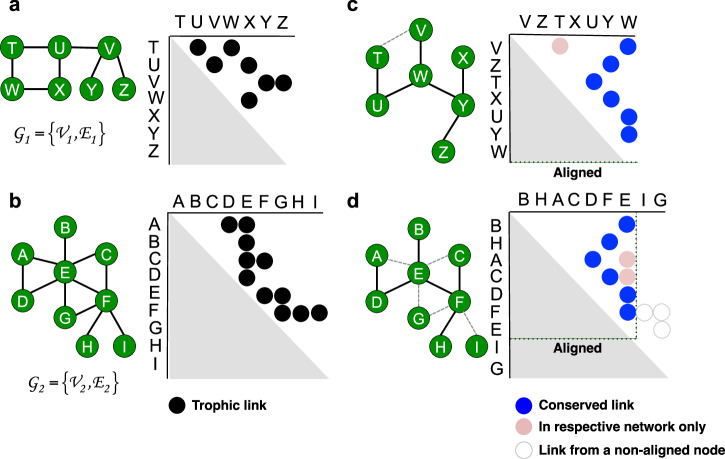
The concept of topological network alignment. **a** Network ${G}_{1}=\left\{{V}_{1},{E}_{1}\right\}$ and **b** Network ${G}_{2}=\left\{{V}_{2},{E}_{2}\right\}$. In both cases, nodes are listed in alphabetical order and only the top half of the adjacency matrix is shown. The two networks appear to be very different in terms of their network diagrams and adjacency matrices, but their network patterns are not directly comparable in their current forms. **c**, **d** Network alignment overlays ${G}_{1}$ on ${G}_{2}$ so as to maximise the similarity of the overall topology. Nodes in the matrix of ${G}_{2}$ are divided into aligned and non-aligned and listed in ascending order of degree within each group, while nodes in the matrix of ${G}_{1}$ have been placed at the same matrix entries as their aligned counterparts to visualise the alignment between nodes. In this example, node V has been assigned to the first entry of its matrix due to its alignment with node B. For the same reason, node Y has been aligned with node F and therefore has been placed at the sixth entry of its matrix. Edges in the network diagrams are divided into those that are conserved (solid) or unique (dashed) to their respective networks. In the matrices, edges are either associated with aligned nodes (filled circles) or non-aligned nodes (not filled circles). The number of conserved edges by the alignment $\left|f\left({E}_{1}\right)\right|$ = 6 (dark circles). The number of edges in the aligned region of ${G}_{1}$ and ${G}_{2}$ are $\left|{E}_{1}\right|$ = 7 (6 dark and 1 light circles) and $E\left|{G}_{2}\left[f\left({V}_{1}\right)\right]\right|$ = 8 (6 dark and 2 light circles) respectively, giving a S^3^ score of 6 / (7 + 8 – 6) = 0.67.

Fresh approaches to ecological network analysis are required because topological patterns that could offer a mechanistic insight into ecosystem responses to environmental change are not generally illustrated by traditional methods, such as whole-network properties like connectance^9,15^. In network theory, it is common to manipulate the entire adjacency matrix of a network (i.e. a square matrix showing connections in a graph) to reveal sub-structures in the topology. For example, global (i.e. whole) network alignment has been an effective tool in bioinformatics for identifying regions that are structurally similar between two networks^16,17^, or in social network analysis for identifying common users in different online communities^18^. In protein-protein interaction networks, species that are linked in terms of evolution are likely to share regions of similarities, and aligning their networks can help compare stages of development and map equivalent functions^16^. The principle of network alignment is to overlay two networks so as to maximise the topological similarity between them^16,19^. Essentially, the process keeps the adjacency matrix of one network and reshuffles the order in which nodes appear in the matrix of the other network until the two adjacency matrices are most similar (Fig. 1); while the actual trophic links among species remain the same. In other words, the technique provides a way to directly compare the actual topology of any two networks by rearranging the matrix presentation of one network to match the other network as much as possible. In ecology, network alignment can offer an effective way in revealing shifts in food web organisation following an environmental stressor. It can also detect structural similarity between ecosystems, providing insights into the mechanics that help maintain ecosystem stability and functioning^20,21^. For example, network alignment has been performed by referring to small local subgraphs to identify species with equivalent functional roles^22^. While global network alignment can help us to better understand the overall structural responses following an environmental change and the re-organisation principles of whole food webs, to the best of our knowledge, this has not yet been exploited in ecology.

Rewiring of trophic links in response to perturbations is likely to be driven by dietary generalism and interconnectedness in food webs. A generalist with a broad range of resource species can largely be regarded as a high degree node with connections to many neighbours, and they can play a central role in governing food web robustness^9,23^. In addition, indirect interactions among generalists who share common resources, which depicts the interconnectivity among neighbours of neighbours in network theory, could provide useful insights into their roles and functions^24^. For example, a generalist with high interconnectivity would indicate that their resources are also consumed by many other generalists. Such highly interconnected species should survive in a competitive environment as they can co-exist with other species that have overlapping niches^25^, indicating their ability to adapt and rewire in the face of environmental perturbations. A well-known metric to gauge interconnectivity in complex networks is the PageRank metric of centrality developed by Google^®^, which ranks the importance of webpages by emphasising those with a high number of links to neighbouring webpages that are also highly linked^26^, and has been used to identify keystone species in ecology^27^. A better understanding of the interconnectivity among species could help reveal the mechanisms underpinning trophic rewiring in food webs.

Climate change causes environmental degradation in many natural habitats, with food web rewiring predicted to be a likely occurrence as species make adaptive changes; however, the extent and distribution of link re-assembly are largely unknown. A common hypothesis in ecology is that abundant generalists with wide diet breaths can redistribute into systems where they were previously absent and forage on new prey^3,28^. Examples include poleward shifts of many species^29^, introducing new trophic links to the associated ecosystems. In addition, there exists a core group of generalist species in food webs, whose ability to adapt their diets and compensate for biodiversity loss could underpin ecosystem responses to perturbations^11^. There is a general consensus that rewiring of species diets is on the rise as more adaptive species switch their diets to adjust for scarce resources in harsher environments^3,30,31^, resulting in higher numbers of weak interactions between consumer and resource species that will help stabilise food webs^32,33^. However, generalists do not respond to stressors in a homogenous way, and their ability to switch or expand their diet should depend on phenotypic variations caused by genetic and environmental factors^34,35^. Thus, a deeper insight into how generalists undergo adaptive dietary changes would enable us to identify critical ecological processes that could help stabilise ecosystem dynamics and restore their functions.

Replicated field experiments are needed to quantify the effects of extreme events on complex food webs without the temporal confounds of exploring changes in a single ecosystem or the spatial confounds of comparing multiple ecosystems with different starting conditions. Here, we examined the extent and distribution of rewiring in highly resolved food webs from a stream mesocosm experiment where benthic communities were subjected to drought or control conditions for two years (see Methods). This long-term, large-scale experiment enabled us to compare the topology of four pairs of control and drought-perturbed food webs using techniques from network alignment, and examine the mechanics that underpin their structural re-organisation and constancy. We hypothesised that high-degree generalist species would play a critical role in maintaining network topology following drought, as they are more likely to undergo adaptive dietary changes due to the fact that they are already able to thrive in a competitive way. To test this, we quantified the level of rewiring among the surviving consumers in the drought webs and assessed if their network properties in the control webs were potential predictors of their trophic plasticity.

## Results and discussion

### Topological constancy in food webs following drought

**Fig. 2:**
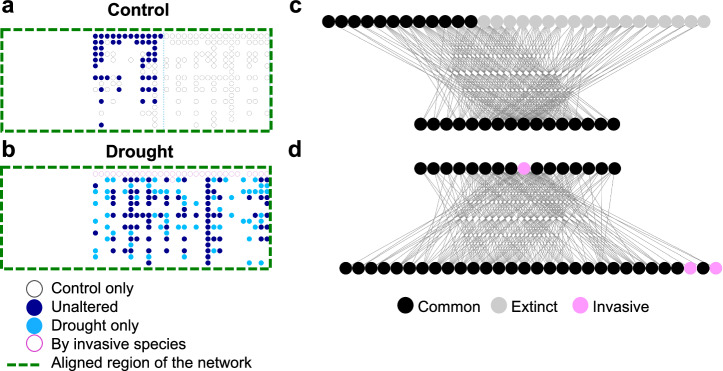
Network alignment reveals similar topological patterns in control-drought pair of webs but formed by different interactions. **a** binary matrix of a control web (Methods), with links that are common to the control and drought pair of webs (dark blue circles) and those have been lost following drought (unfilled grey circles). Only the top halves of the aligned regions are shown (Supplementary Fig. 1a, b). **b** binary matrix of the respective drought web, with rewired links (light blue circles) and those by invasive consumers (unfilled pink circles). Network alignment overlaid the drought matrix with that of the control to maximise similarity in their topologies. Extensive dietary changes among consumers following drought have collectively altered the interactions that formed the aligned network patterns (Supplementary Fig. 1c, d). **c- d** simplified trophic diagrams of (**a**) and (**b**) respectively, whereby consumers are in the top row and resources are in the bottom row. Species included those who survived (dark circles) or went extinct (grey circles), and invasive species (pink circles). Both consumers and resources are arranged in descending order of degree from the centre. The pattern of the aligned control web (**c**) shows a wide range of consumers feeding on a smaller range of resources species; whilst the pattern of the drought web (**d**) is formed by a much smaller range of consumers feeding over a wider range of resource species (Supplementary Fig 1e,f).

Drought greatly perturbed the organisation of links in food webs, despite the previously reported invariance in connectance between the control and drought pairs of webs^36^. On average, 92% of the species in the drought food webs were also found in the control, but only 60% of the trophic links were common to the control (Supplementary Table 1). When we overlaid the adjacency matrices of manipulated webs on their control counterparts and measured their similarity through network alignment (Fig. 1, Methods), drought-impaired food webs had very similar topological organisation to their control counterparts (Fig. 2a, b; Supplementary Fig. 1), with an S^3^ score of 0.80 ± 0.02 (mean ± SD), i.e. 80% similarity. Comparison of control webs to randomised drought webs gave an S^3^ score of 0.49 ± 0.01 and a mean z-score > 30 (Supplementary Table 2), indicating the empirical similarity in topological organisation was dramatically higher than should occur by chance. This high level of resemblance in topology between control and manipulated pairs of webs (compared to the absolute proportion of links retained) implies that there is topological inertia in food web structure whereby organisation patterns have been maintained following drought.

### Re-organisation of trophic links in food webs

Comparisons between the aligned matrices of each pair of control and drought webs revealed systematic link re-assembly following drought, with dietary shifts among consumers restoring a large proportion of the topological configuration (Fig. 2a, b; Supplementary Fig. 2-5; Supplementary Table 3–6). In particular, the topological patterns in the drought webs resembled the most densely connected part of the control webs whereby the connectance of the aligned subgraphs was consistently higher than the whole food webs (Supplementary Table 7). The biotic interactions that constitute these regions of the control webs were associated with a large number of consumers feeding on just a few resources (Fig. 2c). In the drought webs, biodiversity loss led to noticeably fewer consumers (13 ± 1.9 less, Fig. 2d, Supplementary Table 8); however, the surviving consumers were feeding on a much wider range of resources (Supplementary Fig. 1d). The newly established links re-balanced the organisation of the drought webs, resulting in the topological constancy we observed (Supplementary Fig. 6). However, the total biomass fluxes from resources to consumers in the drought webs were generally less than the aligned regions of the control webs (Supplementary Fig. 7; Supplementary Table 9), which implies that ecosystem functioning was reduced despite the compensatory re-organisation of interaction links among species. These collective changes in the link patterns of consumers in the drought webs could be related to the concept of aggregate rewiring^3^, whereby species with similar traits could respond to a stressor en masse by exhibiting an overall shift in their behaviour, which often leads to major rewiring of whole food webs^37,38^. These findings reflect a top-down compensatory effect whereby the surviving consumers capitalise on the loss of other consumers through dietary expansion. The establishment of many new links collectively restored network topology, preserving the robustness of the food web, as observed previously^11^.

### Trophic plasticity in species directly altered their network properties

**Fig. 3:**
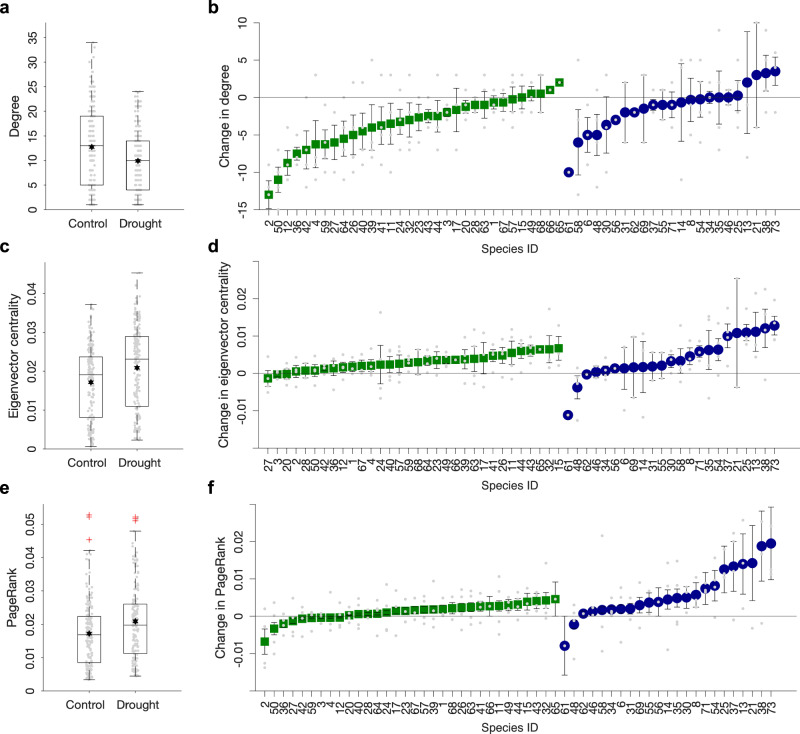
Drought increases interconnectedness among species. **a, c, e** Network- and **b, d, f** node-level differences in (**a, b**) degree, (**c, d**) eigenvector centrality, and (**e, f**) PageRank between control and drought food webs among resource species (green squares, i.e. those without any resources) and consumer species (blue circles, i.e. those with resources which can be with or without consumers themselves). Boxplot showing the mean (hexagram), median (centre lines), and outliners (red crosses), with edges and whiskers representing the 25% and 75% quartiles, and the smallest and largest values of the data respectively, with *n* = 181 for each metric in (**a, c, e**). Species within a group (resource or consumer) are ordered by ascending order of each metric, with *n* = the number of replicates a given species has been recorded in (**b, d, f**). See Table 1 for statistical comparisons between treatments. Only species ID are shown in the x-axes, see Supplementary Table 10 for the reference list of species.

We examined more closely how the network properties among the surviving species changed following the rewiring of trophic links. We found that degree decreased considerably following drought (Fig. 3a, b, Supplementary Table 10), particularly among resources (Table 1), reflecting a reduction in consumption pressure. However, network metrics that are more indicative of interconnectivity among species, such as eigenvector centrality and PageRank (Fig. 3c–f), increased among consumers after drought (Table 1). These findings indicate that consumers have stronger but more distributed influence on the overall dynamics and robustness in the food webs^39^, as they typically have a higher level of interspecific competition by sharing more common resources following environmental perturbation^40^. We found that generalists, such as *Tubificidae* and *Gammarus pulex*, primarily undergo an expansion of diet by widening their consumption of primary producers (Supplementary Fig. 8). Temporal dynamics of abundance measured monthly over a 2-year period indicated that, despite this dietary flexibility under drought conditions, these taxa experienced persistent reductions in abundance compared to control conditions^41^.

**Table 1:** Drought altered species network properties

All species	DF	F-value	*p*-value
Degree	178	51.34	< 0.0001
Eigenvector centrality	178	66.92	< 0.0001
PageRank	178	31.75	< 0.0001
Consumer species			
Degree	66	2.01	0.1609
Eigenvector centrality	66	26.44	< 0.0001
PageRank	66	32.99	< 0.0001
Resource species			
Degree	111	76.30	< 0.0001
Eigenvector centrality	111	50.33	< 0.0001
PageRank	111	4.94	0.0283

The degree, eigenvector centrality, and PageRank of survived species were compared between control and drought webs using linear mixed effects models with species identity nested within paired web identity as the random effect. Subsequently, the survived species were sub-divided into consumer species and resource species. Degrees of freedom (DF), F-values, and *p*-values from the model outputs are shown in the table.

**Fig. 4:**
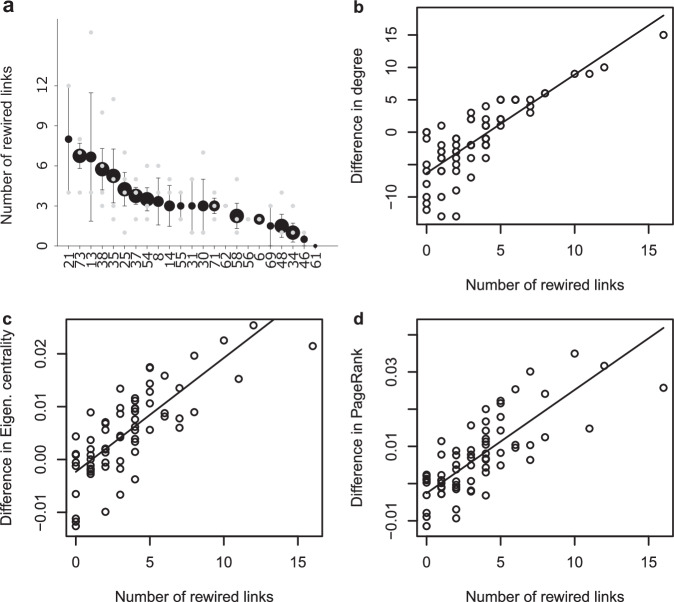
Rewiring alters network properties of species. **a** The mean number of rewired links among the survived consumer species (marker size indicates the number of replicates in which the species was present, *n*= the number of replicates a given species has been recorded). Error bars indicate standard error of the mean. The greater the number of rewired links among species, the greater the changes in node-level properties in the drought webs, including: (**b**) degree (*F*_*1,44*_ = 139.8, *P* < 0.0001, *r*^*2*^ = 0.74; *y* = -6.3235 + 1.5205*x*), (**c**) eigenve**c**tor centrality (*F*_*1,62*_ = 111.1, *P* < 0.0001, *r*^*2*^ = 0.71; *y* = -0.0023 + 0.0022*x*), and (**d**) PageRank (*F*_*1,64*_ = 121.9, *P* < 0.0001, *r*^*2*^ = 0.76; *y* = -0.0026 + 0.0028*x*). Note that the degrees of freedom are based on Satterthwaite’s method, with *n* = 67 for each metric.

Generally, the level of rewiring increased with the level of species turnover (Supplementary Fig. 9), indicating that there is a direct link between the disturbance magnitude and the compensatory re-assembly required to buffer these effects. Of the 23 surviving consumer species, all but one exhibited rewiring, with the level of rewiring varying among species and among replicates (Fig. 4a). When examining if changes in network properties of species were related to trophic plasticity, we found that the magnitude of change in degree, eigenvector centrality, and PageRank all linearly increased with the number of rewired links (Fig. 4b–d). This shows that dietary shifts among consumer species increased their network centrality by broadening their range of resources to those also shared with other generalists. These compensatory mechanics among the surviving consumers collectively reshaped the overall structural configuration, manifesting into topological constancy in drought-impaired food webs.

### Specialist species proportionally rewired the most

**Fig. 5:**
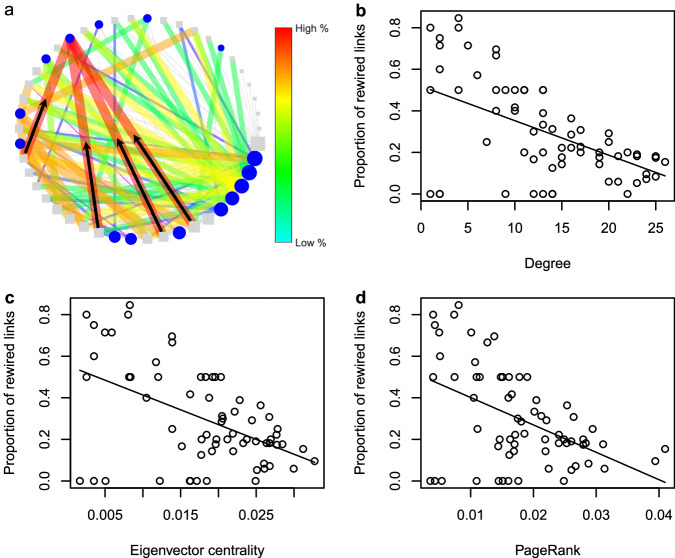
Adaptability among poorly connected species governs persistence in the face of drought. **a** Specialist species with narrower diets were found to have rewired proportionally more than generalist species. Consumer (blue circles) and resource species (grey squares) are placed in ascending order of degree, indicated by node size. Links are divided into unaltered links from the control web (unweighted grey lines) and rewired links that are only found in the drought web (weighted colour lines), both colour and weight indicate the proportion of rewired links of a given species. An example of a low degree consumer with a high proportion of rewired links is shown, with the transfer of energy fluxes from new resource species indicated by black arrows. See Supplementary Fig. 10 for all webs. The proportion of links that were rewired in the drought webs decreased with: (**b**) degree (*F*_*1,31*_ = 21.65, *P* < 0.0001, *r*^*2*^ = 0.47; *y* = 0.5186 - 0.0166*x*), (**c**) eigenvector centrality (*F*_*1,29*_ = 23.07, *P* < 0.0001, *r*^*2*^ = 0.55; *y* = 0.5559 - 14.25*x*), and (**d**) PageRank (*F*_*1,33*_ = 22.46, *P* < 0.0001, *r*^*2*^ = 0.55; *y* = 0.5331 – 13.18*x*). Note that the degrees of freedom are based on Satterthwaite’s method, with *n* = 67 for each metric.

We examined which characteristics of consumer species in the control food webs determined the level of rewiring they would undergo after drought. In contrast to our hypothesis, we found that virtually all consumers rewired (Supplementary Fig. 10) and that specialists with more restricted diets proportionally rewired more than generalists (Fig. 5a). Overall, the proportion of rewired links per consumer species in the drought treatment was negatively correlated with degree, eigenvector centrality, and PageRank (Fig. 5b–d), which means that species with the most specialised diets exhibited the most adaptive changes, in relative terms. Generally, the surviving specialists all expanded their diets to mitigate the effects of drought, revealing a level of trophic plasticity that has not been commonly reported. We found that some specialists vastly expanded their diets within the same functional groups of resources following drought (e.g. *Cricotopus sp*.), while others even started feeding on entirely new functional groups (e.g. *Macropelopia sp*. on herbivores and *Cryptochironomus sp*. on primary producers; Supplementary Fig. 11). Monthly abundance data over a 2-year period from the experiment indicated that this unexpected level of dietary change may have helped these taxa maintain their abundance in the face of drought^41^. These findings show that dietary specialists also play an important role in reconfiguring food web link organisation and re-distributing energy fluxes. Primary extinctions can alter the availability of resources through release from competition or top-down control^42^, thus affecting foraging parameters such as encounter and consumption rates^43^ and associated dietary composition^44^. How these complex interactions are inter-related is yet to be fully investigated, but our results demonstrate that system-wide rewiring among both surviving generalists and specialists is critical in compensating for the effects of drought and fostering topological resilience of food webs.

### Discussion and broadening perspective

We used network alignment to show that rewiring in drought-impaired food webs, manifested by system-wide changes in the diet of the surviving consumers, preserved the overall network topology, which may be crucial for stability and maintenance of biodiversity^44,45^. Existing metrics in ecological network analysis, such as connectance or linkage density, only deliver a simplistic and partial understanding on the overall network organisation^9,15^. Our approach provides an expansive way to directly gauge the level of structure invariance (and variance) in ecological networks, and reveal the underlying compensatory mechanics that govern ecosystem reassembly. More specifically, we uncovered that trophic plasticity, even among facultative dietary specialists, may thus play a key role in mitigating the effects of environmental stressors and rebalancing the composition and dynamics of ecosystems, and help confer food web robustness^11^. Drought is promoted by a warming climate, which may alter metabolic demands and thus food consumption rates among consumer species^38^. Our study shows that ecosystem functioning was reduced despite rewiring, echoing previous findings on substantial adjustment of biomass fluxes following drought, with increasing biomass fluxes among small taxa and reductions for larger ones^14^.

Furthermore, we showed that interconnectedness among species was higher following drought, implying a higher level of niche overlap among species. This was likely caused by habitat degradation triggered by drought, which in turn led to greater competition for food sources among the surviving consumers^40^. This agrees with optimal foraging theory, which predicts trophic niche broadening as a result of perturbations as resources become scarcer and consumers include less profitable resources in their diets^46,47^.

Our results demonstrate that the mechanics underpinning ecosystem re-assembly do not only rely on dietary generalists, which is in contrary to common ecological assumptions on their governing role on topological rewiring^48,49^. While generalists have a greater ability to adapt and dietary changes were common among them, only relatively small changes to their diets were sufficient to buffer the effects of drought. In contrast, the specialist species that survived underwent major shifts in their diet which moved them towards a more generalised approach^50,51^, and this was achieved by broadening diet both within existing functional groups of species and into other groups. It should be noted that this may only be possible for facultative specialists, i.e. those that choose to have a narrower diet under benign environmental conditions^52^, as opposed to obligate specialists who may not have the capacity to alter their diet (but see ref.^53^). These findings reveal that trophic plasticity among species with a seemingly restricted niche not only plays a critical role in their own survival, but may also be key to the re-assembly of trophic links and associated dynamics of ecosystems.

It is worth pointing out that only topological rewiring was examined here, but the way in which interaction strengths have been altered by drought have not been fully examined. The strength and weight of trophic links are likely to be unevenly distributed across an ecological network, and the availability and distribution of resources could alter carbon fluxes to consumers differently following drought^54,55^. Hence, future studies should assess new metrics of network alignment that incorporate link weights to enhance our understanding of how drought-induced changes in networks alter ecosystem functioning^56,57^. It should also be noted that our exploration of network alignment here is from a single snapshot in time at the end of a 2-year experiment. Whilst abundance data was collected monthly to explore changes in temporal dynamics in response to the drought treatments^41^, there was insufficient dietary information to adequately describe temporal changes in food web structure. Thus, further investigations should quantify dietary shifts through time in response to environmental stressors like drought to explore the temporal dynamics of network alignment.

Ecological communities are increasingly subjected to environmental and anthropogenic pressures, and understanding how these communities respond to and mitigate the effects of climatic stressors is central to their safeguarding. Adaptive dietary shift is likely to be one key mechanism for species to persist in the face of biodiversity loss and greater physiological stress under the current rapidly changing climate. Hence, a better understanding of trophic plasticity in species will greatly enhance our ability to identify key ecological processes that preserve ecosystem functions in the face of global environmental change, which will help direct conservation and biomonitoring efforts.

## Methods

Samples were collected from experimental habitats housed within the grounds of the Freshwater Biological Association River Laboratory, Dorset, UK, with the full permission of the Freshwater Biological Association director and staff. Macroinvertebrates are collected routinely as part of biomonitoring / research programmes across the UK and no special permission is required to collect them.

### Dataset

We analysed existing food web data from a previous outdoor stream mesocosm experiment in which benthic communities subjected to a drought treatment (a six-day drying event conducted monthly for two years) were compared with those from undisturbed controls. The experiment ran for 24 months (March 2000–February 2002) in four blocks of two linear outdoor stream channel mesocosms at the Freshwater Biological Association River Laboratory, UK, which were filled to 20 cm depth with stony beds and shallow subsurface sediment^36,58^. Stream water was fed into all channels with a 2-month colonization period, followed by an intermittent flow regime (6 days of flow cassation per month) being applied to one mesocosm in each block, with the second channel in each block acting as a control. The drought treatment simulated the repeated, patchy dewatering that occurs during severe supra-seasonal droughts. There were four replicates of each treatment sampled at the end of the experiment, resulting in eight food webs in total. Food webs were constructed from direct observation of feeding links via dissected gut contents of all 3643 individual invertebrates collected at the end of the experiment. Prey were always identified to the lowest taxonomic resolution possible, usually species, even when their abundances were low. Thus, there was no systematic bias towards dietary generalists or specialists in the treatment of the gut content data. These exceptionally well-resolved webs encompassed 783 pairwise trophic interactions among 74 trophic elements, consisting of detrital resources, primary producers, and a taxonomically diverse array of invertebrate consumers (Supplementary Table 3). We used the trophic basis of production to quantify directly-observed feeding links from resources to consumers for each mesocosm community^59^, as biomass fluxes in g m^-2^ yr^-1^. Comparison of the experimental control food webs to data collected for 82 natural river food webs showed the mesocosms contained realistic food webs, with consistent and similar size structures suggesting that patterns of energy flux between experimental consumers and resources were good analogues of those in natural ecosystems^60^.

### Network alignment

In bioinformatics, structural patterns in protein-protein interaction networks or neural networks are often representative of certain functions. Network alignment has been widely used to compare different snapshots of these networks to determine how the interactions among a group of proteins or connectomes evolve over time^61^; or to compare patterns of interactions between species to identify common functions^17^. Here, we employed network alignment using Magna++ to search for the best superimposition of topology between each pair of control and drought food webs^16,19^ to assess the level of topological constancy. In general, the search space for such superimposition is extremely large due to the vast number of possible mappings between nodes, and techniques for network alignment rely on heuristics to speed up and optimise a search. Magna++ generates a population of alignments, and uses genetic algorithms to simulate an evolutionary process whereby only alignments that conserve the most links are selected in each generation until the maximum similarity between the two networks is reached (Fig. 1). More formally, given two undirected and unweighted networks ${G}_{1}=\left\{{V}_{1},{E}_{1}\right\}$ and ${G}_{2}=\left\{{V}_{2},{E}_{2}\right\}$, where ${V}_{n}$ is the set of nodes (species) and ${E}_{n}$ is the set of edges (trophic links) in network $n$ respectively, the method finds an alignment $f:{V}_{1}\to {V}_{2}$ between nodes in the two networks that would maximise a cost function $Q\left({G}_{1},{G}_{2},{f}\right)$. We refer to the Symmetric Substructure Score^19^, S^3^, as the cost function which gauges the accuracy of an alignment by taking both ${G}_{1}$ and ${G}_{2}$ into consideration. This metric has been shown to provide a more accurate assessment as it penalises misalignments of edges from a dense region to a sparse region, and vice versa:

$${S}^{3}=\frac{\left|f\left({E}_{1}\right)\right|}{\left|{E}_{1}\right|+\left|E\left({G}_{2}\left[f\left({V}_{1}\right)\right]\right)\right|-\left|f\left({E}_{1}\right)\right|}$$

where $\left|f\left({E}_{1}\right)\right|$ is the number of edges conserved by the alignment $f$, $\left|{E}_{1}\right|$ is the number of edges in ${G}_{1}$, ${G}_{2}\left[f\left({V}_{1}\right)\right]$ is the subgraph formed by nodes in ${G}_{2}$ that have been aligned to nodes in ${G}_{1}$ and $E\left|{G}_{2}\left[f\left({V}_{1}\right)\right]\right|$ is the number of edges within this subgraph.

Undirected adjacency matrices of control and manipulated webs were used in the network alignment. Magna++ can either be optimised by conserving nodes or a balance between nodes and edges. We used the former in this study because it generally obtained better S^3^ scores. The default settings of Magna++ were used with population size = 15,000, number of generations = 2000, fraction of elite members = 0.5, and number of threads = 4. To check the robustness of the approach, a given food web was aligned with itself and all the species were found to be matched with themselves, indicating that the algorithm was working correctly^62^. Given the stochastic nature of the algorithm, results were averaged over 30 runs as in previous studies^61^.

### Null models

To test the significant of S^3^ scores between the alignment of each pair of webs, we generated 30 randomised versions of the drought webs and aligned them with their respective control webs. The randomised drought webs were obtained by reshuffling trophic links while preserving the overall degree sequence^63^. We calculated the z-score for each pair of alignment using $z=\frac{x-\mu }{\sigma }$, whereby

$x$ is the S^3^ score between a control and drought web pair, while $\mu$ and $\sigma$ are the mean and standard deviation of the S^3^ scores between the control and the 30 randomised drought webs.

### Aligned binary matrices

Binary matrices of the control and drought food webs contained trophic links between consumer and resource species, and were aligned so that the similarity between the topology of each pair of webs could be easily compared. In a control web, species were divided into two groups: aligned and non-aligned, i.e. nodes from the control web that were or were not matched with nodes from the drought web, respectively. Within the aligned and non-aligned groups, species were ordered into those which survived or went extinct in the drought web. Hence, we had the following four sub-groups: aligned survived, aligned extinct, non-aligned survived, and non-aligned extinct. Within each of these sub-groups, species were categorised into two broad functional groups of resource and consumer species. Resource species were those that do not consume any live organisms or materials such as detritus, decomposers, or primary producers. Detritus included amorphous detritus and plant fragments which did not have recorded body mass, and they formed the first two entries of the matrices. Decomposers included fungal spores and *Hyphomycete fungal hyphae*; while primary producers included the rest of the resource species which were ordered according to species body mass. Consumer invertebrate species included detritivores, herbivores, and predators, and each sub-group was also ordered according to species body mass. Species in the corresponding drought matrix do not follow this ordering but were placed in the same matrix entries as their aligned counterparts in the control (Fig. 1c, d). For example, if we assume species B in a drought web has been aligned with species A in the corresponding control web, and species A has been placed in the 10th entry of the control matrix by the aforementioned ordering method, then species B would be placed in the 10th entry of the drought matrix.

### Network metrics

We referred to the commonly used food web property of degree (number of trophic links to other species) to assess connectedness in the network analysis. In addition, we examined eigenvector centrality and PageRank to evaluate interconnectedness among species. Eigenvector centrality has been widely used to measure the influence of a node in a complex network whereby edges associated with high degree nodes would be considered more useful than those with low degree nodes^64^. The eigenvector centrality is based on the eigenvalue, meaning that the value of a node is related to the value of its neighbours, and thus if their neighbours have higher degrees, the eigenvector centrality of the node will be higher. PageRank is a variant of eigenvector centrality which is the first algorithm used by Google^®^ to index webpages in their search engine^26^. The PageRank algorithm measures the importance of each node within a given web, based on the number of incoming links and the importance of the corresponding source nodes. The algorithm assigns scores to all the nodes in a network and updates these scores in a recursive manner until an equilibrium is reached. The ‘centrality’ function in MATLAB^®^ R2018 was used to calculate both eigenvector centrality and PageRank. All results were averaged over the four replicates of each treatment in the experiment.

### Statistics and reproducibility

To compare network metrics between aligned groups of species in control and drought pairs of food webs, linear mixed effects models were performed on degree, eigenvector centrality, and PageRank as response variables and treatment as a categorical explanatory variable with two levels (control and drought), with a random intercept for species identity nested within each pair of webs. Linear mixed effects models were also performed on the difference in degree, eigenvector centrality, and PageRank among all survived consumer species as response variables with the number of rewired links as a continuous explanatory variable, with crossed random intercepts for web pair identity and species identity. Finally, linear mixed effects models were performed on the proportion of rewired links as the response variable and degree, eigenvector centrality, and PageRank as continuous explanatory variables, with crossed random intercepts for web pair identity and species identity.

### Reporting summary

Further information on research design is available in the Nature Portfolio Reporting Summary linked to this article.

## Supplementary information

Supplementary Information

Description of Additional Supplementary Materials

Supplementary Data 1

Supplementary Data 2

Supplementary Software

Reporting Summary

## Data Availability

Data are available as part of Supplementary Information and the version of Magna++ used is available at Zenodo repository^65^ (Supplementary Data 1-2, Supplementary Software). Correspondence and requests for materials should be addressed to A.M.
