# Supplementary material for: Network rewiring conserves the topology of drought-impaired food webs
Source: Commun Biol. 2025 Nov 24;8:1641. doi: 10.1038/s42003-025-09035-2 (PMC12644991; doi:10.1038/s42003-025-09035-2)
Supplement: Supplementary file 1 — Supplementary Information [file 42003_2025_9035_MOESM1_ESM.pdf]

Network rewiring conserves the topology of drought-impaired food webs

Supplementary Figures and Tables

Supplementary Table 1. Rewiring observed in all webs. For each drought web, the proportions of surviving species and unaltered links, which are nodes and links that are common to both webs, are listed. The proportion of rewired links in each drought web was calculated by referring to the surviving species only (Exc. Invasion), and by referring to all links including species that invaded the drought webs (All). The Jaccard Index among nodes and links between each web pair are also provided, together with the overall mean values and standard deviations (S.D.) across all webs.

	Unaltered in drought web		Rewiring in drought web		The Jaccard index between web pairs	
web	*Nodes*	*Links*	*Exc. Invasion*	*All*	*Nodes*	*Links*
1	0.91	0.58	0.32	0.42	0.32	0.62
2	0.87	0.48	0.36	0.52	0.42	0.79
3	0.92	0.69	0.12	0.31	0.31	0.67
4	0.96	0.66	0.17	0.34	0.25	0.66
Mean	0.92	0.60	0.24	0.40	0.33	0.69
S.D.	0.04	0.09	0.12	0.09	0.07	0.07

Supplementary Table 2. High level of topological similarity between control and drought web pairs. For each pair of webs, the mean S^3 scores from 30 runs are shown, together with the overall mean values and standard deviations (S.D.) across all webs. The mean S^3 scores between control and 30 randomised drought webs are also shown by comparing the results from the empirical webs with the null model. Note that the z-score indicates the number of standard deviations that the empirical webs lie from the mean of the randomisations.

	Empirical webs		Empirical control and randomised drought webs		
Food web pair	S^3	S.D.	S^3	S.D.	z-score
1	0.79	0.01	0.47	0.01	32
2	0.83	0.01	0.51	0.01	32
3	0.81	0.02	0.48	0.01	33
4	0.78	0.01	0.49	0.01	25
Mean	0.80	0.02	0.49	0.01	30.5

Supplementary Table 3. Species alignment in control and drought pair of webs 1. Species in the control web are grouped into aligned and non-aligned groups. Within a group, survived species (non-shaded) are listed first in ascending order of body mass, followed by extinct species (*shaded*) in the same order. Species in the drought web are listed against their counterpart species in the control web based on the network alignment results. An asterisk (*) denotes an invaded species.

Web pair 1	Species in control web	Trophic group		Aligned species in drought web	Trophic group
Aligned	Amorphus detritus	Detritus		Tubificidae	Detritivore
	Plant fragments	Detritus		*Cricotopus sp.*	Herbivore
	Fungal spores	Decomposer		*Heterotrissocladius sp.*	Detritivore
	Hyphomycete fungal hyphae	Decomposer		*Tinodes waeneri*	Herbivore
	Chrococcus minor	Primary producer		*Microtendipes sp.*	Herbivore
	Psammothidium lauenburgianum	Primary producer		*Cryptochironomus sp.*	Herbivore
	Gomphonema olivaceum	Primary producer		Naididae	Detritivore
	Amphora pediculus	Primary producer		*Ephemera danica*	Detritivore
	Planothidium lanceolatum	Primary producer		*Oulimnius tuberculatus*	Detritivore
	Encyonema minutum	Primary producer		*Limnius volckmari*	Detritivore
	Gongrosira incrustans	Primary producer		*Gammarus pulex*	Detritivore
	Melosira varians	Primary producer		*Radix balthica*	Herbivore
	Cocconeis placentula	Primary producer		*Baetis sp.*	Herbivore
	Navicula menisculus	Primary producer		*Pisidium sp.*	Detritivore
	Nitzschia dissipata	Primary producer	*	*Eiseniella tetraedra*	Detritivore
	Nitzschia perminuta	Primary producer		*Macropelopia sp.*	Predator
	Rhoicosphenia abbreviata	Primary producer		*Potamopyrgus antipodarum*	Herbivore
	Navicula tripunctata	Primary producer		Simuliidae	Detritivore
	Navicula lanceolata	Primary producer		*Hydropsyche sp.*	Predator
	Spirulina sp.	Primary producer	*	*Synorthocladius sp.*	Herbivore
	Naididae	Detritivore		*Chrococcus minor*	Primary producer
	Oulimnius tuberculatus	Detritivore	*	*Staurosirella leptostauron*	Primary producer
	Heterotrissocladius sp.	Detritivore		*Gongrosira incrustans*	Primary producer
	Simuliidae	Detritivore		*Cocconeis placentula*	Primary producer
	Tubificidae	Detritivore		*Nitzschia perminuta*	Primary producer
	Pisidium sp.	Detritivore		Fungal spores	Decomposer
	Gammarus pulex	Detritivore		*Amphora pediculus*	Primary producer

	Ephemera danica	Detritivore		*Rhoicosphenia abbreviata*	Primary producer
	Cricotopus sp.	Herbivore		*Synedra ulna*	Primary producer
	Cryptochironomus sp.	Herbivore		*Surirella minuta*	Primary producer
	Microtendipes sp.	Herbivore		Amorphus detritus	Detritus
	Tinodes waeneri	Herbivore		*Navicula tripunctata*	Primary producer
	Baetis sp.	Herbivore		*Nitzschia dissipata*	Primary producer
	Potamopyrgus antipodarum	Herbivore		*Navicula menisculus*	Primary producer
	Radix balthica	Herbivore		*Gomphonema olivaceum*	Primary producer
	Hydropsyche sp.	Predator		*Navicula gregaria*	Primary producer
	Amphora ovalis	Primary producer	*	*Sialis lutaria*	Predator
	Cymatopleura solea	Primary producer	*	*Gyrosigma sp.*	Primary producer
	Ostracoda	Detritivore		*Encyonema minutum*	Primary producer
	Leuctra geniculata	Detritivore		*Psammothidium lauenburgianum*	Primary producer
	Polypedilum sp.	Detritivore		*Spirulina sp.*	Primary producer
	Elmis aenea	Detritivore		*Planothidium lanceolatum*	Primary producer
	Asellus aquaticus	Detritivore		*Melosira varians*	Primary producer
	Sericostoma personatum	Detritivore		*Navicula lanceolata*	Primary producer
	Athripsodes sp.	Herbivore		*Staurosira elliptica*	Primary producer
	Brachycentrus subnubilus	Herbivore		Plant fragments	Detritus
	Ancylus fluviatilis	Herbivore		*Hyphomycete fungal hyphae*	Decomposer
Non-aligned	*Staurosira elliptica*	Primary producer			
	Surirella minuta	Primary producer			
	Navicula gregaria	Primary producer			
	Synedra ulna	Primary producer			
	Limnius volckmari	Detritivore			
	Macropelopia sp.	Predator			
	Fragilaria vaucheriae	Primary producer			
	Algal cysts	Primary producer			
	Diatoma vulgare	Primary producer			
	Brychius elevatus	Herbivore			
	Polycentropus flavomaculatus	Predator			
	Erpobdella octoculata	Predator			

Supplementary Table 4. Species alignment in control and drought pair of webs 2. Species in the control web are grouped into aligned and non-aligned groups. Within a group, survived species (non-shaded) are listed first in ascending order of body mass, followed by extinct species (*shaded*) in the same order. Species in the drought web are listed against their counterpart species in the control web based on the network alignment results. An asterisk (*) denotes an invaded species.

Web pair 2	Species in control web	Trophic group	Aligned species in drought web	Trophic group
Aligned	Amorphus detritus	Detritus	* *Tipula montium*	Detritivore
	Plant fragments	Detritus	Naididae	Detritivore
	Hyphomycete fungal hyphae	Decomposer	*Macropelopia sp.*	Predator
	Staurosira elliptica	Primary producer	*Ephemera danica*	Detritivore
	Gomphonema olivaceum	Primary producer	*Gammarus pulex*	Detritivore
	Amphora pediculus	Primary producer	*Potamopyrgus antipodarum*	Herbivore
	Encyonema minutum	Primary producer	*Pisidium sp.*	Detritivore
	Cocconeis placentula	Primary producer	*Microtendipes sp.*	Herbivore
	Navicula gregaria	Primary producer	*Cryptochironomus sp.*	Herbivore
	Rhoicosphenia abbreviata	Primary producer	Tubificidae	Detritivore
	Algal cysts	Primary producer	*Synorthocladius sp.*	Herbivore
	Navicula tripunctata	Primary producer	*Radix balthica*	Herbivore
	Amphora ovalis	Primary producer	*Limnius volckmari*	Detritivore
	Navicula lanceolata	Primary producer	*Tinodes waeneri*	Herbivore
	Cymatopleura solea	Primary producer	*Procladius sp.*	Herbivore
	Spirulina sp.	Primary producer	*Asellus aquaticus*	Detritivore
	Tubificidae	Detritivore	*Staurosira elliptica*	Primary producer
	Gammarus pulex	Detritivore	*Hyphomycete fungal hyphae*	Decomposer
	Pisidium sp.	Detritivore	*Navicula lanceolata*	Primary producer
	Asellus aquaticus	Detritivore	*Chrococcus minor*	Primary producer
	Ephemera danica	Detritivore	Algal cysts	Primary producer
	Synorthocladius sp.	Herbivore	*Amphora ovalis*	Primary producer
	Procladius sp.	Herbivore	*Diatoma vulgare*	Primary producer
	Cryptochironomus sp.	Herbivore	*Surirella minuta*	Primary producer
	Tinodes waeneri	Herbivore	*Navicula tripunctata*	Primary producer

	Potamopyrgus antipodarum	Herbivore	*Nitzschia perminuta*	Primary producer
	Radix balthica	Herbivore	*Amorphus detritus*	Detritus
	Macropelopia sp.	Predator	*Planothidium lanceolatum*	Primary producer
	Heterotrissocladius sp.	Detritivore	*Navicula gregaria*	Primary producer
	Polypedilum sp.	Detritivore	* *Surirella brebissonii*	Primary producer
	Simuliidae	Detritivore	*Navicula menisculus*	Primary producer
	Elmis aenea	Detritivore	*Nitzschia dissipata*	Primary producer
	Oulimnius tuberculatus	Detritivore	* *Gyrosigma sp.*	Primary producer
	Sericostoma personatum	Detritivore	*Gomphonema olivaceum*	Primary producer
	Cricotopus sp.	Herbivore	*Cymatopleura solea*	Primary producer
	Athripsodes sp.	Herbivore	*Spirulina sp.*	Primary producer
	Brachycentrus subnubilus	Herbivore	Plant fragments	Detritus
	Brychius elevatus	Herbivore	*Fragilaria vaucheriae*	Primary producer
	Ancylus fluviatilis	Herbivore	*Melosira varians*	Primary producer
	Valvata piscinalis	Herbivore	*Cocconeis placentula*	Primary producer
	Theodoxus fluviatilis	Herbivore	*Amphora pediculus*	Primary producer
	Pentaneura sp.	Predator	*Psammothidium lauenburgianum*	Primary producer
	Polycentropus flavomaculatus	Predator	*Rhoicosphenia abbreviata*	Primary producer
	Hydropsyche sp.	Predator	Fungal spores	Decomposer
	Haliplus lineatocollis	Predator	*Encyonema minutum*	Primary producer
	Sialis lutaria	Predator	*Gongrosira incrustans*	Primary producer
Non-aligned	Fungal spores	Decomposer		
	Chrococcus minor	Primary producer		
	Psammothidium lauenburgianum	Primary producer		
	Planothidium lanceolatum	Primary producer		
	Gongrosira incrustans	Primary producer		
	Melosira varians	Primary producer		
	Navicula menisculus	Primary producer		
	Surirella minuta	Primary producer		
	Nitzschia dissipata	Primary producer		

Nitzschia perminuta	Primary producer
Fragilaria vaucheriae	Primary producer
Diatoma vulgare	Primary producer
Naididae	Detritivore
Limnius volckmari	Detritivore
Microtendipes sp.	Herbivore
Staurosirella leptostauron	Primary producer
Erpobdella octoculata	Predator

Supplementary Table 5. Species alignment in control and drought pair of webs 3. Species in the control web are grouped into aligned and non-aligned groups. Within a group, survived species (non-shaded) are listed first in ascending order of body mass, followed by extinct species (*shaded*) in the same order. Species in the drought web are listed against their counterpart species in the control web based on the network alignment results. An asterisk (*) denotes an invaded species.

Web pair 3	Species in control web	Trophic group		Aligned species in drought web	Trophic group
**Aligned**	Amorphus detritus	Detritus		Tubificidae	Detritivore
	Plant fragments	Detritus		*Microtendipes sp.*	Herbivore
	Fungal spores	Decomposer		*Pisidium sp.*	Detritivore
	Chrococcus minor	Primary producer		*Baetis sp.*	Herbivore
	Staurosira elliptica	Primary producer		*Cricotopus sp.*	Herbivore
	Psammothidium lauenburgianum	Primary producer	*	*Cryptochironomus sp.*	Herbivore
	Gomphonema olivaceum	Primary producer		*Asellus aquaticus*	Detritivore
	Amphora pediculus	Primary producer	*	*Eiseniella tetraedra*	Detritivore
	Staurosirella leptostauron	Primary producer	*	*Cymbella lanceolata*	Primary producer
	Encyonema minutum	Primary producer		*Encyonema minutum*	Primary producer
	Melosira varians	Primary producer		*Heterotrissocladius sp.*	Detritivore
	Navicula menisculus	Primary producer		*Tinodes waeneri*	Herbivore
	Navicula gregaria	Primary producer		Naididae	Detritivore
	Nitzschia dissipata	Primary producer	*	*Brachycentrus subnubilus*	Herbivore
	Nitzschia perminuta	Primary producer		*Radix balthica*	Herbivore
	Rhoicosphenia abbreviata	Primary producer		*Gammarus pulex*	Detritivore
	Algal cysts	Primary producer		*Macropelopia sp.*	Predator
	Navicula tripunctata	Primary producer		*Potamopyrgus antipodarum*	Herbivore
	Amphora ovalis	Primary producer		*Limnius volckmari*	Detritivore
	Gyrosigma sp.	Primary producer		*Sialis lutaria*	Predator
	Naididae	Detritivore		*Melosira varians*	Primary producer
	Tubificidae	Detritivore		*Amphora pediculus*	Primary producer
	Pisidium sp.	Detritivore		Fungal spores	Decomposer
	Limnius volckmari	Detritivore		*Cymatopleura solea*	Primary producer
	Asellus aquaticus	Detritivore		*Navicula tripunctata*	Primary producer
	Gammarus pulex	Detritivore		*Cocconeis placentula*	Primary producer
	Tinodes waeneri	Herbivore		*Navicula lanceolata*	Primary producer

	Cricotopus sp.	Herbivore		*Navicula gregaria*	Primary producer
	Microtendipes sp.	Herbivore		*Gomphonema olivaceum*	Primary producer
	Baetis sp.	Herbivore		*Nitzschia perminuta*	Primary producer
	Potamopyrgus antipodarum	Herbivore		*Chrococcus minor*	Primary producer
	Radix balthica	Herbivore		Plant fragments	Detritus
	Macropelopia sp.	Predator		Algal cysts	Primary producer
	Surirella minuta	Primary producer		*Psammothidium lauenburgianum*	Primary producer
	Ostracoda	Detritivore	*	*Surirella brebissonii*	Primary producer
	Elmis aenea	Detritivore		*Planothidium lanceolatum*	Primary producer
	Oulimnius tuberculatus	Detritivore		*Diatoma vulgare*	Primary producer
	Simuliidae	Detritivore		*Navicula menisculus*	Primary producer
	Ephemera danica	Detritivore		*Gongrosira incrustans*	Primary producer
	Tipula montium	Detritivore		*Nitzschia dissipata*	Primary producer
	Synorthocladius sp.	Herbivore		*Fragilaria vaucheriae*	Primary producer
	Athripsodes sp.	Herbivore		*Hyphomycete fungal hyphae*	Decomposer
	Ancylus fluviatilis	Herbivore		*Rhoicosphenia abbreviata*	Primary producer
	Valvata piscinalis	Herbivore		*Amorphus detritus*	Detritus
	Pentaneura sp.	Predator		*Spirulina sp.*	Primary producer
	Polycentropus flavomaculatus	Predator		*Staurosirella leptostauron*	Primary producer
	Haliplus lineatocollis	Predator		*Gyrosigma sp.*	Primary producer
	Hydropsyche sp.	Predator		*Staurosira elliptica*	Primary producer
	Platambus maculatus	Predator		*Amphora ovalis*	Primary producer
**Non-aligned**	*Hyphomycete fungal hyphae*	Decomposer			
	Planothidium lanceolatum	Primary producer			
	Gongrosira incrustans	Primary producer			
	Cocconeis placentula	Primary producer			
	Fragilaria vaucheriae	Primary producer			
	Diatoma vulgare	Primary producer			
	Navicula lanceolata	Primary producer			
	Cymatopleura solea	Primary producer			
	Spirulina sp.	Primary producer			
	Heterotrissocladius sp.	Detritivore			

Sialis lutaria	Predator
Erpobdella octoculata	Predator

Supplementary Table 6. Species alignment in control and drought pair of webs 4. Species in the control web are grouped into aligned and non-aligned groups. Within a group, survived species (non-shaded) are listed first in ascending order of body mass, followed by extinct species (*shaded*) in the same order. Species in the drought web are listed against their counterpart species in the control web based on the network alignment results. An asterisk (*) denotes an invaded species.

Web pair 4	Species in control web	Trophic group	Aligned species in drought web	Trophic group
Aligned	Amorphus detritus	Detritus	* *Tipula montium*	Detritivore
	Plant fragments	Detritus	*Microtendipes sp.*	Herbivore
	Fungal spores	Decomposer	*Baetis sp.*	Herbivore
	Hyphomycete fungal hyphae	Decomposer	*Heterotrissocladius sp.*	Detritivore
	Chrococcus minor	Primary producer	*Tubificidae*	Detritivore
	Staurosira elliptica	Primary producer	*Prodiamesa olivacea*	Detritivore
	Psammothidium lauenburgianum	Primary producer	*Spirulina sp.*	Primary producer
	Gomphonema olivaceum	Primary producer	* *Tinodes waeneri*	Herbivore
	Amphora pediculus	Primary producer	*Radix balthica*	Herbivore
	Planothidium lanceolatum	Primary producer	*Oulimnius tuberculatus*	Detritivore
	Encyonema minutum	Primary producer	*Macropelopia sp.*	Predator
	Melosira varians	Primary producer	*Gammarus pulex*	Detritivore
	Cocconeis placentula	Primary producer	*Cricotopus sp.*	Herbivore
	Navicula menisculus	Primary producer	*Potamopyrgus antipodarum*	Herbivore
	Surirella minuta	Primary producer	*Pisidium sp.*	Detritivore
	Surirella brebissonii	Primary producer	*Limnius volckmari*	Detritivore
	Nitzschia perminuta	Primary producer	*Hydropsyche sp.*	Predator
	Fragilaria vaucheriae	Primary producer	*Procladius sp.*	Herbivore
	Rhoicosphenia abbreviata	Primary producer	*Asellus aquaticus*	Detritivore
	Algal cysts	Primary producer	*Cryptochironomus sp.*	Herbivore
	Navicula tripunctata	Primary producer	Naididae	Detritivore
	Amphora ovalis	Primary producer	*Synorthocladius sp.*	Herbivore
	Synedra ulna	Primary producer	*Synedra ulna*	Primary producer
	Gyrosigma sp.	Primary producer	*Surirella brebissonii*	Primary producer
	Oulimnius tuberculatus	Detritivore	*Encyonema minutum*	Primary producer
	Naididae	Detritivore	*Cocconeis placentula*	Primary producer
	Heterotrissocladius sp.	Detritivore	*Hyphomycete fungal hyphae*	Decomposer

	Tubificidae	Detritivore	Plant fragments	Detritus
	Limnius volckmari	Detritivore	*Gyrosigma sp.*	Primary producer
	Prodiamesa olivacea	Detritivore	*Rhoicosphenia abbreviata*	Primary producer
	Asellus aquaticus	Detritivore	*Staurosira elliptica*	Primary producer
	Pisidium sp.	Detritivore	*Navicula lanceolata*	Primary producer
	Procladius sp.	Herbivore	*Diatoma vulgare*	Primary producer
	Synorthocladius sp.	Herbivore	*Fragilaria vaucheriae*	Primary producer
	Cryptochironomus sp.	Herbivore	*Gongrosira incrustans*	Primary producer
	Baetis sp.	Herbivore	*Surirella minuta*	Primary producer
	Microtendipes sp.	Herbivore	Amorphus detritus	Detritus
	Potamopyrgus antipodarum	Herbivore	*Navicula tripunctata*	Primary producer
	Radix balthica	Herbivore	*Amphora pediculus*	Primary producer
	Macropelopia sp.	Predator	*Nitzschia perminuta*	Primary producer
	Hydropsyche sp.	Predator	*Navicula gregaria*	Primary producer
	Cymbella lanceolata	Primary producer	Algal cysts	Primary producer
	Polypedilum sp.	Detritivore	Fungal spores	Decomposer
	Elmis aenea	Detritivore	*Amphora ovalis*	Primary producer
	Sericostoma personatum	Detritivore	*Gomphonema olivaceum*	Primary producer
	Ephemera danica	Detritivore	*Navicula menisculus*	Primary producer
	Athripsodes sp.	Herbivore	*Planothidium lanceolatum*	Primary producer
	Ancylus fluviatilis	Herbivore	*Melosira varians*	Primary producer
	Valvata piscinalis	Herbivore	*Nitzschia dissipata*	Primary producer
	Theodoxus fluviatilis	Herbivore	*Chrococcus minor*	Primary producer
	Pentaneura sp.	Predator	*Cymatopleura solea*	Primary producer
	Haliplus lineatocollis	Predator	*Psammothidium lauenburgianum*	Primary producer
Non-aligned	*Gongrosira incrustans*	Primary producer		
	Navicula gregaria	Primary producer		
	Nitzschia dissipata	Primary producer		
	Diatoma vulgare	Primary producer		
	Navicula lanceolata	Primary producer		
	Cymatopleura solea	Primary producer		
	Spirulina sp.	Primary producer		

Gammarus pulex	Detritivore
Cricotopus sp.	Herbivore
Ostracoda	Detritivore
Polycentropus flavomaculatus	Predator
Sialis lutaria	Predator
Erpobdella octoculata	Predator

Supplementary Table 7. **Trophic plasticity restored densely connected regions in food webs.** For each pair of webs, the connectance of the control, aligned subgraph in control and drought are shown, together with mean values and standard deviations (S.D.) across all webs.

Food web pair	Control	Aligned control subgraph	Drought
1	0.0862	0.1123	0.1254
2	0.0756	0.0893	0.0879
3	0.0806	0.1125	0.0991
4	0.0710	0.0991	0.0973
Mean	0.0783	0.1033	0.1024
S.D.	0.0065	0.0112	0.0161

Supplementary Table 8. The number of consumers and resources in each web.

Food web pair	Control		Drought	
	Consumers	Resources	Consumers	Resources
1	30	29	21	26
2	34	29	16	30
3	31	30	18	31
4	33	32	21	31
Mean	32	30	19	29.5
S.D.	1.8257	1.1442	2.4495	2.3805

Supplementary Table 9. **Reduction in biomass fluxes following drought.** For each pair of webs, the total biomass fluxes measured in g m^{-2} y^{-1} from resources to consumers of the aligned subgraph in control and drought, and the percentages in reduction are shown, together with mean values across all webs.

Food web pair	Aligned control subgraph	Drought	% in reduction
1	153.43	60.07	60.85
2	121.39	33.66	72.27
3	40.17	32.24	19.74
4	55.95	32.70	41.55
Mean	92.74	39.67	48.60

Supplementary Table 10. Species ID and their names.

1	*Algal cysts*
2	Amorphus detritus
3	*Amphora ovalis*
4	*Amphora pediculus*
5	*Ancylus fluviatilis*
6	*Asellus aquaticus*
7	*Athripsodes sp.*
8	*Baetis sp.*
9	*Brachycentrus subnubilus*
10	*Brychius elevatus*
11	*Chrococcus minor*
12	*Cocconeis placentula*
13	*Cricotopus sp.*
14	*Cryptochironomus sp.*
15	*Cymatopleura solea*
16	*Cymbella lanceolata*
17	*Diatoma vulgare*
19	*Elmis aenea*
20	*Encyonema minutum*
21	*Ephemera danica*
22	*Erpobdella octoculata*
23	*Fragilaria vaucheriae*
24	Fungal spores
25	*Gammarus pulex*
26	*Gomphonema olivaceum*
27	*Gongrosira incrustans*
28	*Gyrosigma sp.*
29	*Haliplus lineatocollis*
30	*Heterotrissocladius sp.*
31	*Hydropsyche sp.*
32	*Hyphomycete fungal hyphae*
33	*Leuctra geniculata*
34	*Limnius volckmari*
35	*Macropelopia sp.*
36	*Melosira varians*
37	*Microtendipes sp.*
38	Naididae
39	*Navicula gregaria*
40	*Navicula lanceolata*
41	*Navicula menisculus*
42	*Navicula tripunctata*
43	*Nitzschia dissipata*
44	*Nitzschia perminuta*
45	*Ostracoda*
46	*Oulimnius tuberculatus*
47	*Pentaneura sp.*
48	*Pisidium sp.*
49	*Planothidium lanceolatum*
50	Plant fragments
51	*Platambus maculatus*
52	*Polycentropus flavomaculatus*
53	*Polypedilum sp.*
54	*Potamopyrgus antipodarum*
55	*Procladius sp.*
56	*Prodiamesa olivacea*
57	*Psammothidium lauenburgianum*
58	*Radix balthica*
59	*Rhoicosphenia abbreviata*
60	*Sericostoma personatum*
61	*Sialis lutaria*
62	Simuliidae
63	*Spirulina sp.*
64	*Staurosira elliptica*
65	*Staurosirella leptostauron*
66	*Surirella brebissonii*
67	*Surirella minuta*
68	*Synedra ulna*
69	*Synorthocladius sp.*
70	*Theodoxus fluviatilis*
71	*Tinodes waeneri*
72	*Tipula montium*
73	Tubificidae
74	*Valvata piscinalis*

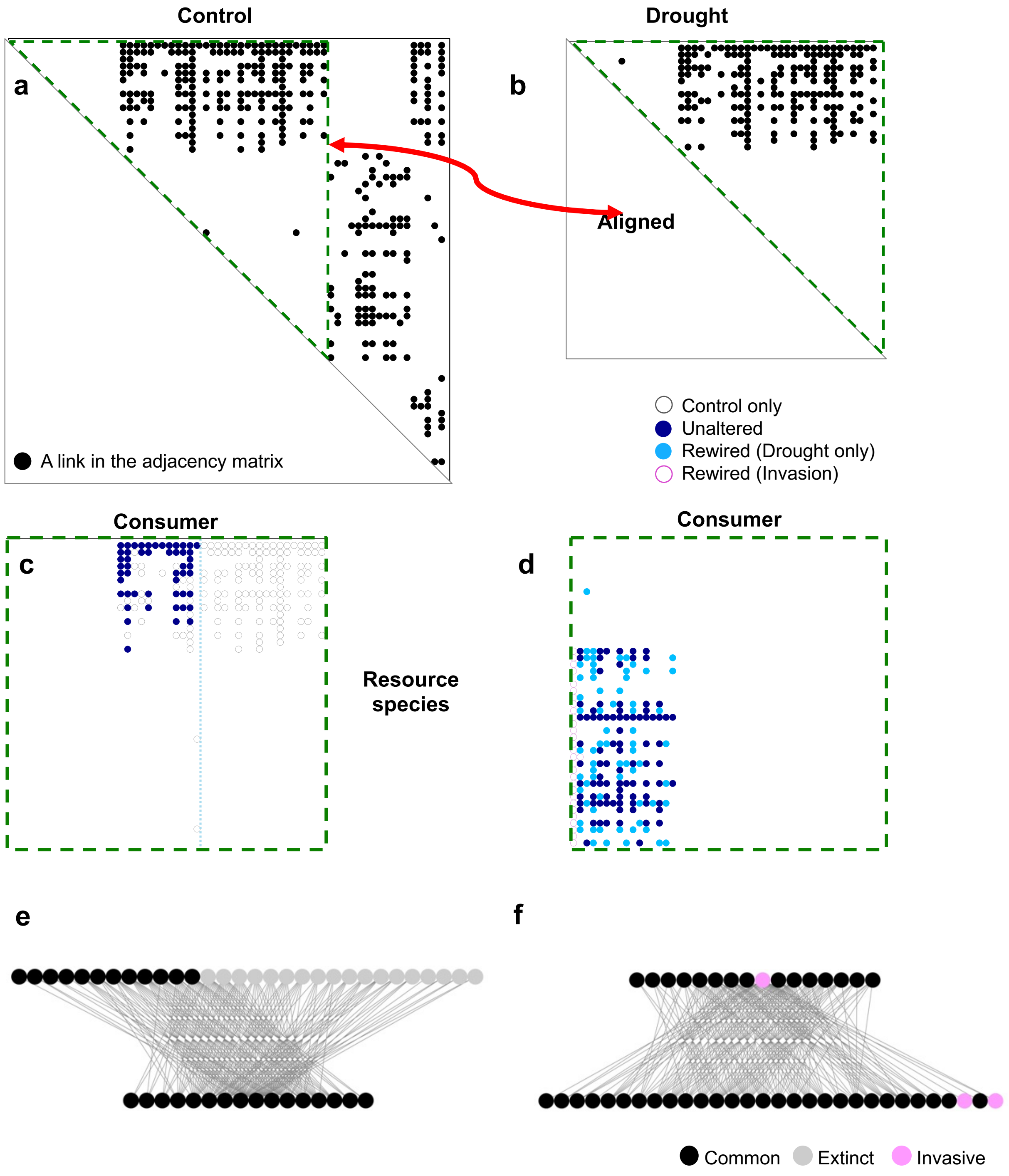

Supplementary Figure 1. Network alignment between a control and drought pair of webs. **a** Undirected adjacency matrix of a control web with undirected links (black circles) whereby species are sorted into aligned and non-aligned (see Methods). Only the top half of the matrix is shown here. b, Undirected adjacency matrices of the correspond drought web. Biodiversity loss following drought means that the drought web has fewer nodes. Here, nodes have been aligned with a corresponding node in the control web (boxed by green dashed line) that maximised the similarity in the topology, revealing structural inertia following drought. **c** The aligned region of the binary matrix of a control web whereby species are placed in the same order as (**a**). A link can either be unaltered by drought (dark blue circles) or in control only (unfilled grey circles), with the latter mostly associated with extinct consumers on the right-hand side of the matrix (marked by dotted vertical line). **d** The aligned

region of the binary matrix of the corresponding drought web whereby species are placed in the same order as (**b**). In additional to unaltered links, rewired links as a result of dietary change (light blue circles) and species invasion (unfilled pink circles) are shown. Here, it can be seen that while the network patterns from the control-drought pair of webs are similar following alignment, the actual interactions and dynamics that made up these patterns are very different. The former is governed by a much wider range of consumer species who fed on a few resource species, while the latter shows a few consumer species feeding on a large number of resources as a result of trophic plasticity. Only one pair of webs is shown here, all webs are shown in (Supplementary Fig. 2-5**). e,f** Simplified trophic diagrams of a control-drought pair of webs (**c,d**) respectively. Consumers and resources are in the top and bottom rows respectively. Species include those who survived (dark circles) or went extinct (grey circles), and invasive species (pink circles). Both consumers and resources are arranged in descending order of degree from the centre. In the aligned region of the control web (**e**), the topological pattern is formed by a large number of consumers over a relatively small range of resources. In the drought web, the pattern is made up by a small number of consumers and a wider range of resources. Only one pair of webs is shown here, all webs are shown in (Supplementary Fig. 6)**.**

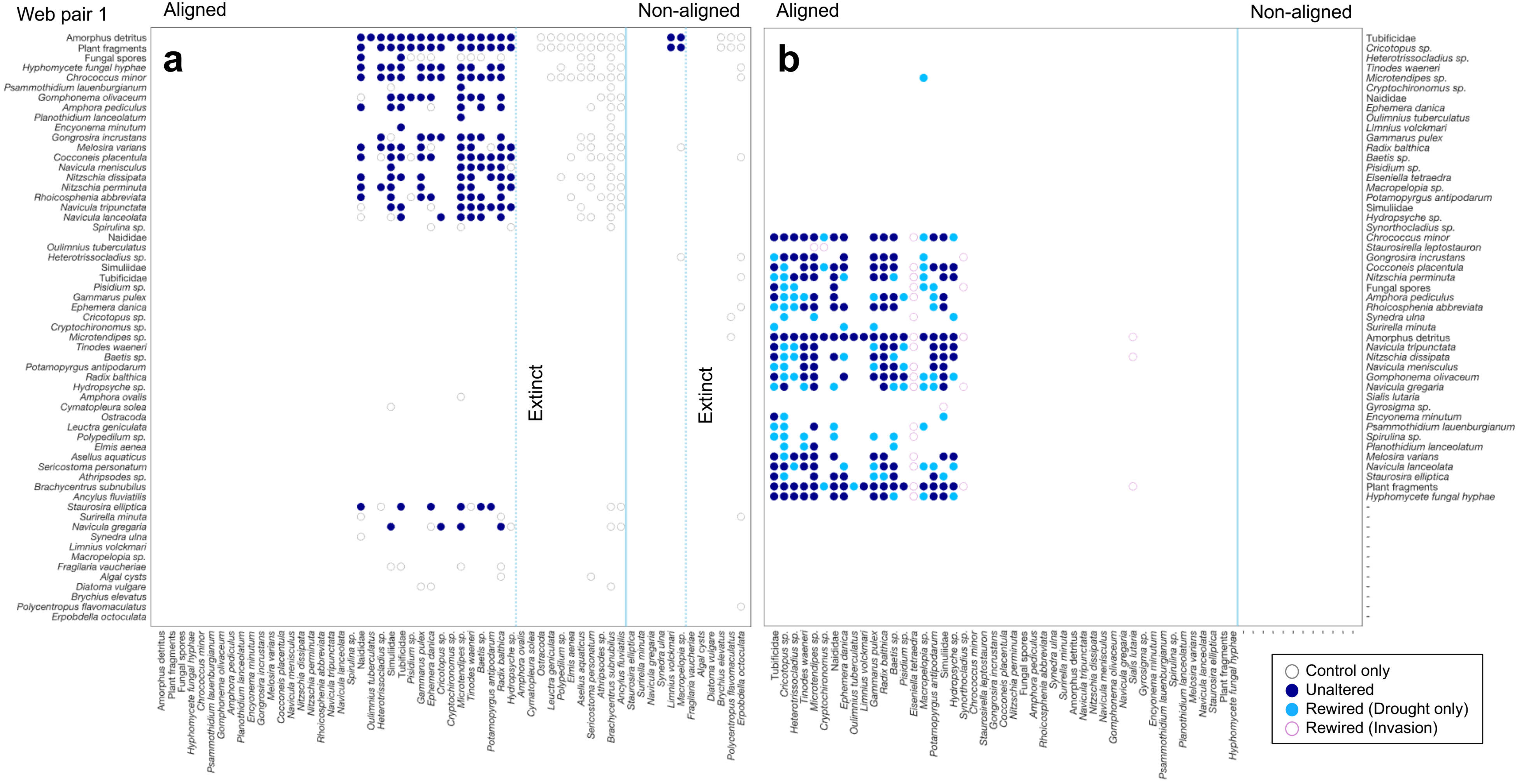

Supplementary Figure 2. Network Alignment between control and drought pair of webs 1. a Sorted binary matrix of the control web. Aligned species are sorted by their trophic groups (resource species followed by consumers, see Methods) and then by ascending order of body mass. Survived species and extinct species are separated by the blue dotted line. Non-aligned species are ordered in the same way. Among the survived species, we observed interactions that are common to both webs (dark filled circles) and those that no longer exist in the drought web (non-filled circles, dark border). **b** Binary matrix of the drought web whereby species have been placed in the same network locations of their aligned species in the control web. New interactions arise through survived species establishing new feeding links (light filled circles), or as a result of invaded species (non-filled circles, light border). Details on the species are listed in Supplementary Table 3.

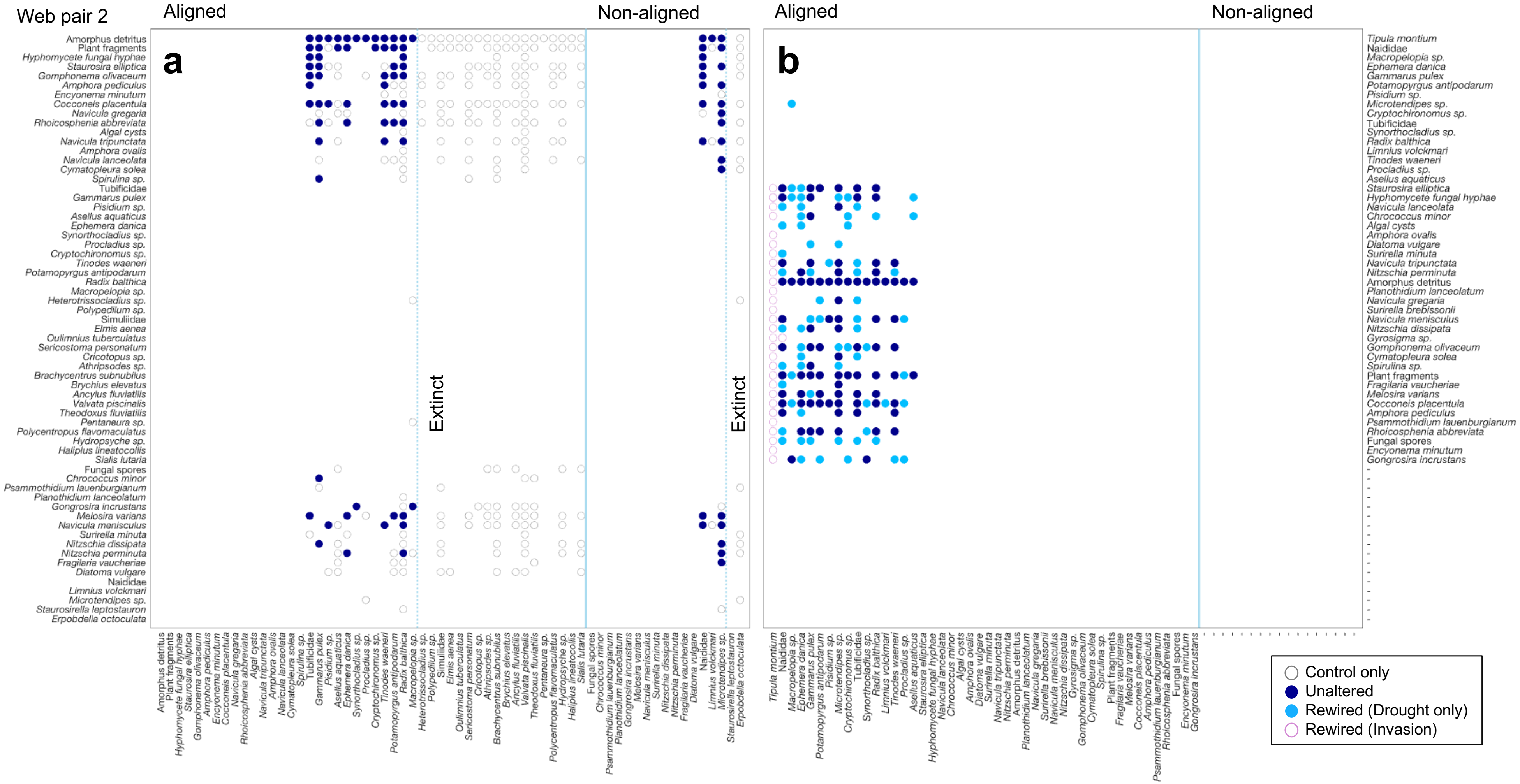

Supplementary Figure 3. Network Alignment between control and drought pair of webs 2. a Sorted binary matrix of the control web. Aligned species are sorted by their trophic groups (resource species followed by consumers, see Methods) and then by ascending order of body mass. Survived species and extinct species are separated by the blue dotted line. Non-aligned species are ordered in the same way. Among the survived species, we observed interactions that are common to both webs (dark filled circles) and those that no longer exist in the drought web (non-filled circles, dark border). **b** Binary matrix of the drought web whereby species have been placed in the same network locations of their aligned species in the control web. New interactions arise through survived species establishing new feeding links (light filled circles), or as a result of invaded species (non-filled circles, light border). Details on the species are listed in Supplementary Table 4.

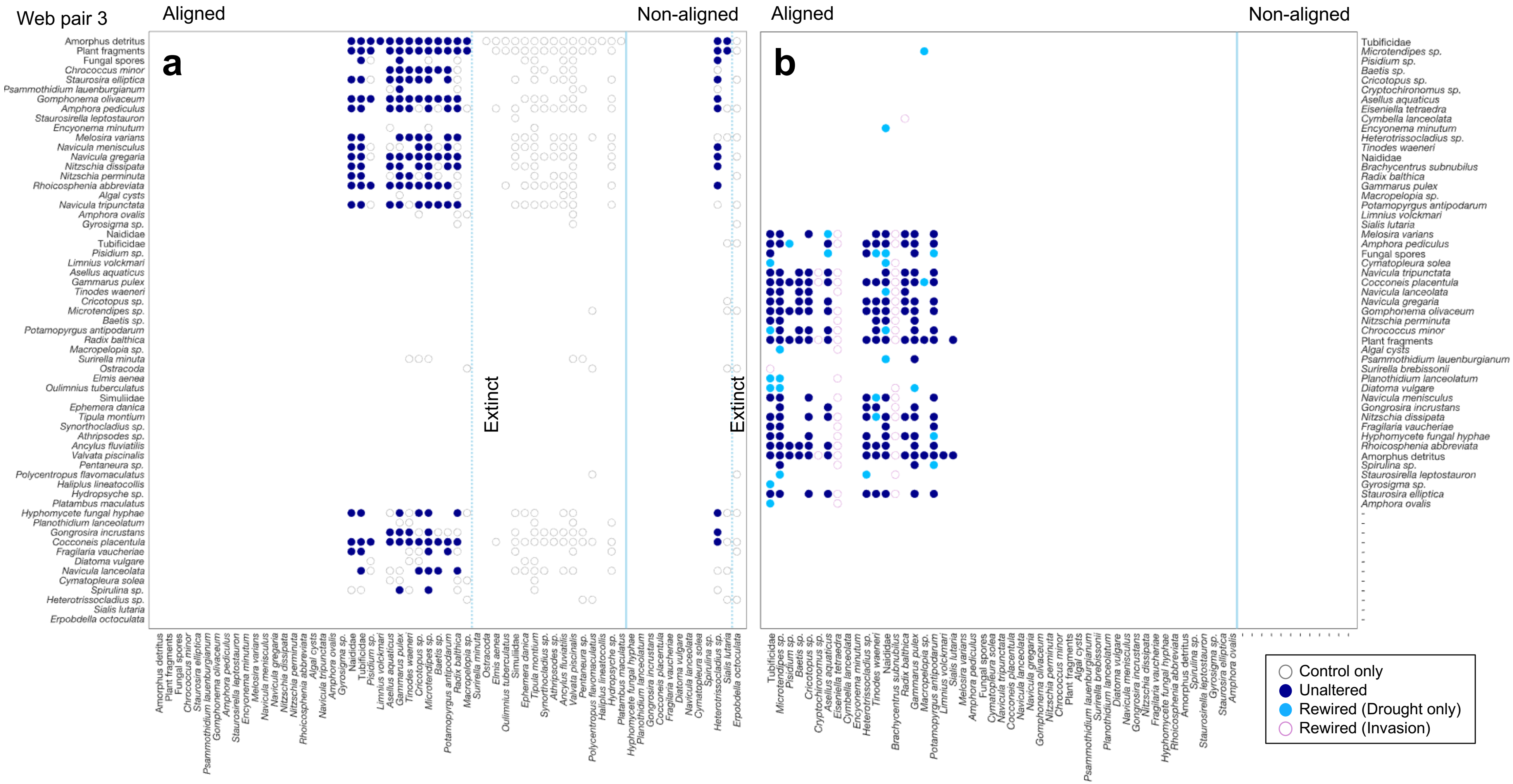

Supplementary Figure 4. Network Alignment between control and drought pair of webs 3. a Sorted binary matrix of the control web. Aligned species are sorted by their trophic groups (resource species followed by consumers, see Methods) and then by ascending order of body mass. Survived species and extinct species are separated by the blue dotted line. Non-aligned species are ordered in the same way. Among the survived species, we observed interactions that are common to both webs (dark filled circles) and those that no longer exist in the drought web (non-filled circles, dark border). **b** Binary matrix of the drought web whereby species have been placed in the same network locations of their aligned species in the control web. New interactions arise through survived species establishing new feeding links (light filled circles), or as a result of invaded species (non-filled circles, light border). Details on the species are listed in Supplementary Table 5.

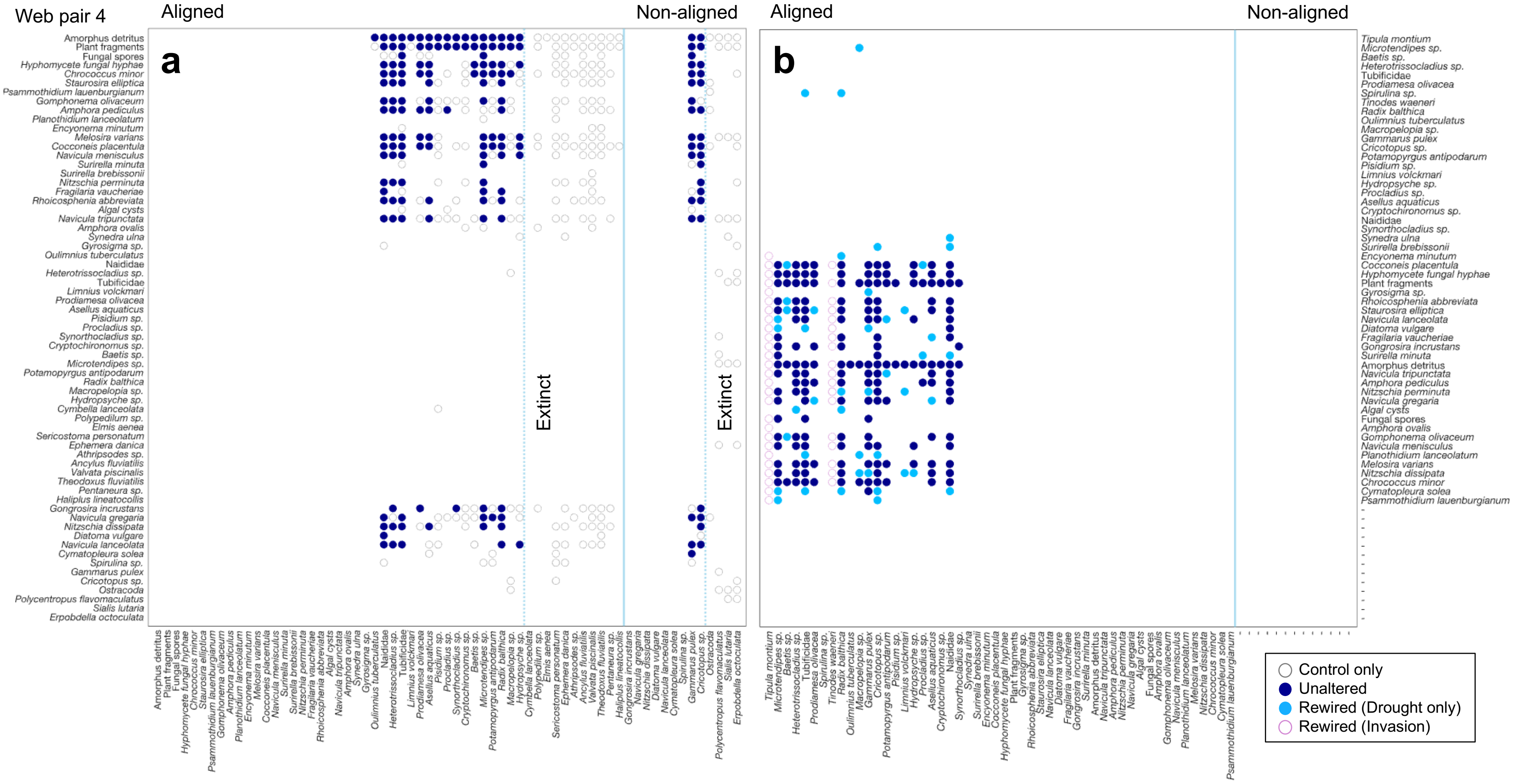

Supplementary Figure 5. Network Alignment between control and drought pair of webs 4. a Sorted binary matrix of the control web. Aligned species are sorted by their trophic groups (resource species followed by consumers, see Methods) and then by ascending order of body mass. Survived species and extinct species are separated by the blue dotted line. Non-aligned species are ordered in the same way. Among the survived species, we observed interactions that are common to both webs (dark filled circles) and those that no longer exist in the drought web (non-filled circles, dark border). **b** Binary matrix of the drought web whereby species have been placed in the same network locations of their aligned species in the control web. New interactions arise through survived species establishing new feeding links (light filled circles), or as a result of invaded species (non-filled circles, light border). Details on the species are listed in Supplementary Table 6.

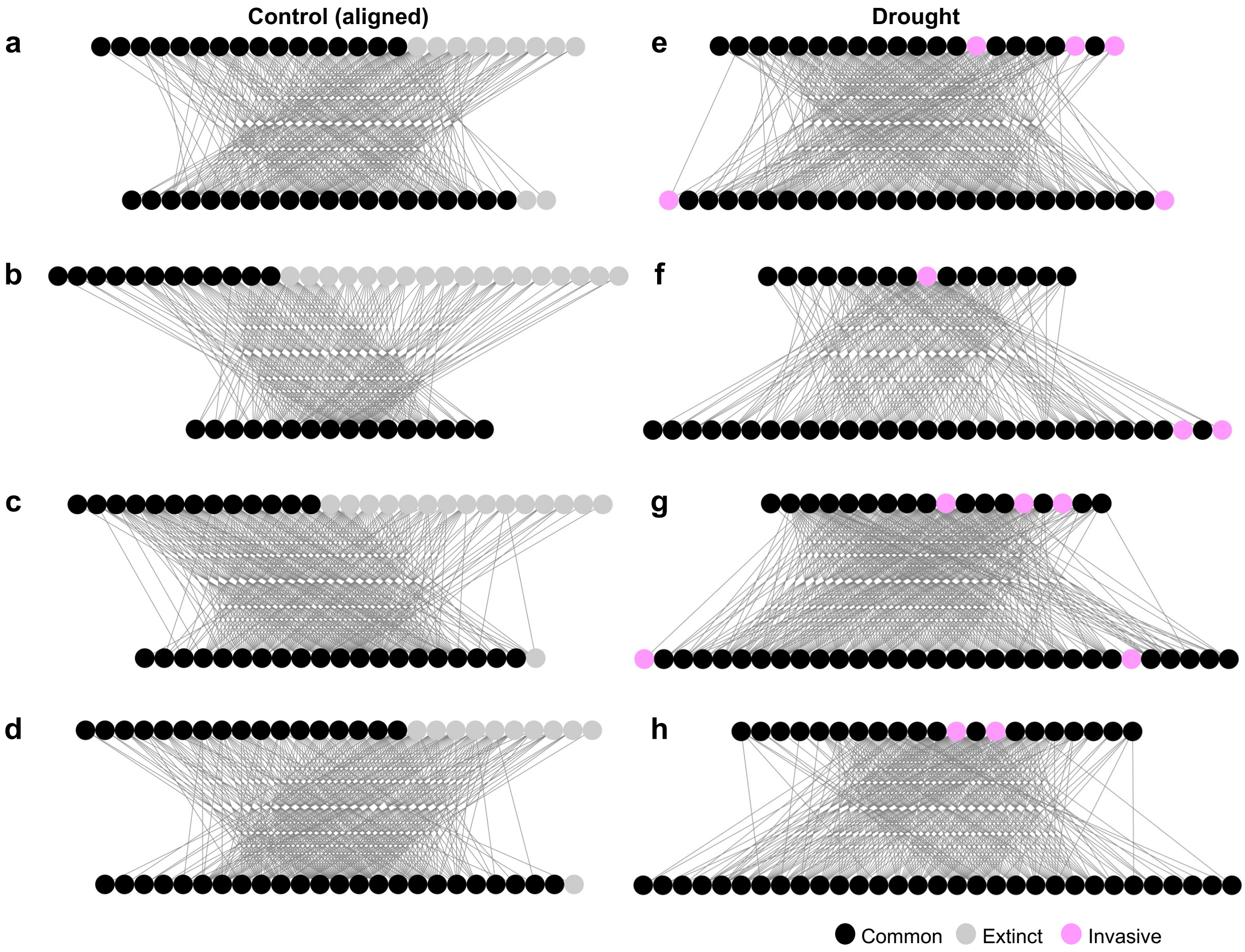

Supplementary Figure 6. Topological patterns in drought webs are manifested by fewer consumers with wider diet breaths. a-h simplified trophic diagrams of the control (a-d) and drought (e-h) pairs of webs, with species who survived drought (dark circles) or went extinct (grey circles), and invasive species (pink circles). Both consumers and resources are arranged in descending order of degree from the centre. The patterns of the aligned control webs (a-d) show a wide range of consumers feeding on a smaller range of resources species; whilst the patterns of the drought webs (e-h) are formed by a much smaller range of consumers feeding over a wider range of resource species.

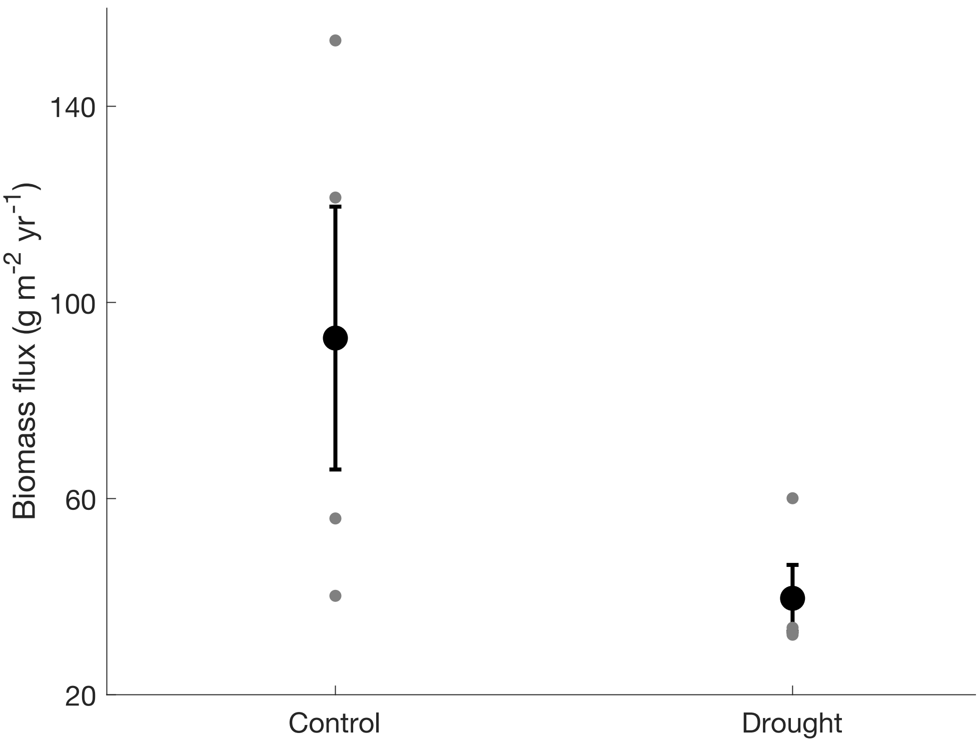

Supplementary Figure 7. Reduction in the total biomass fluxes following drought. The total biomass fluxes measured in g m^{-2} y^{-1} from resources to consumers in drought webs and in their aligned control counterparts, with an average of 49% reduction of biomass fluxes in the former (*two-tailed paired t*-test, *d.f.* = 3, p = 0.09; Supplementary Table 9). Error bars indicate standard error of the mean.

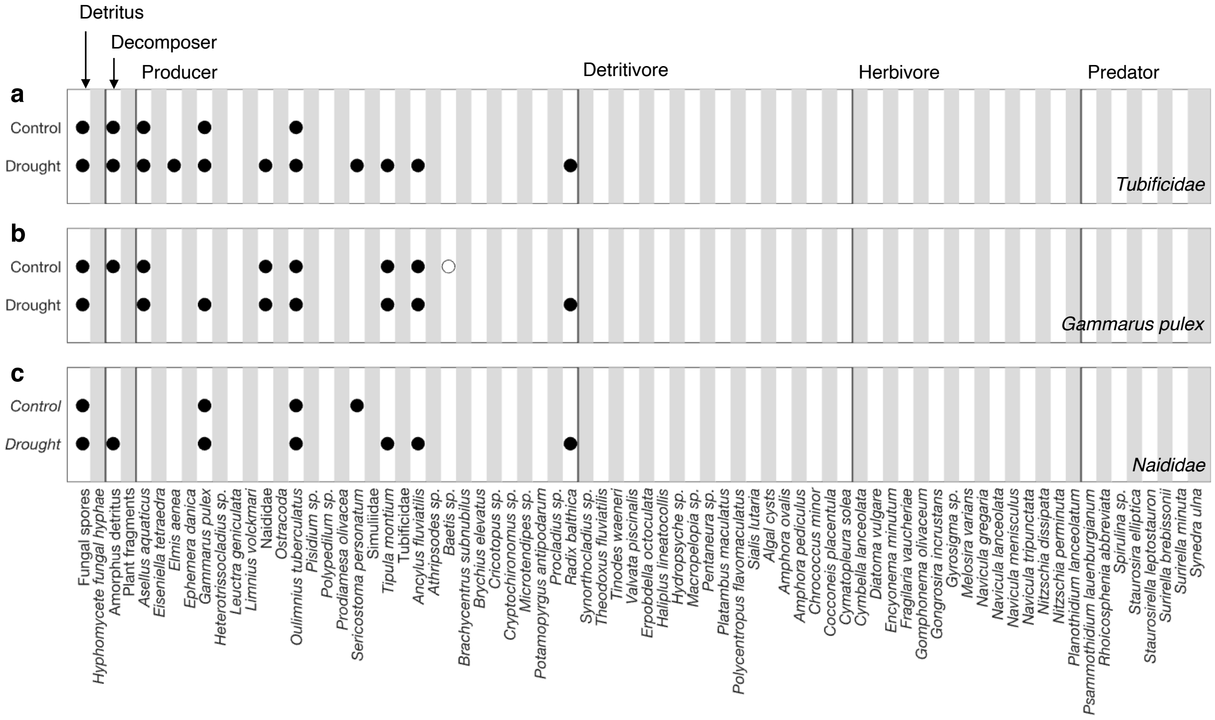

Supplementary Figure 8. Dietary shifts among the highly interconnected generalists. a-c *Tubificidae*, *Gammarus pulex* and *Naididae* respectively. Species on the x-axis are categorised into functional groups and then ordered by increasing order of body mass. Resources (filled circles) of a given generalists are shown in a pair of control and drought webs, with those who are extinct following drought indicated (not filled circles).

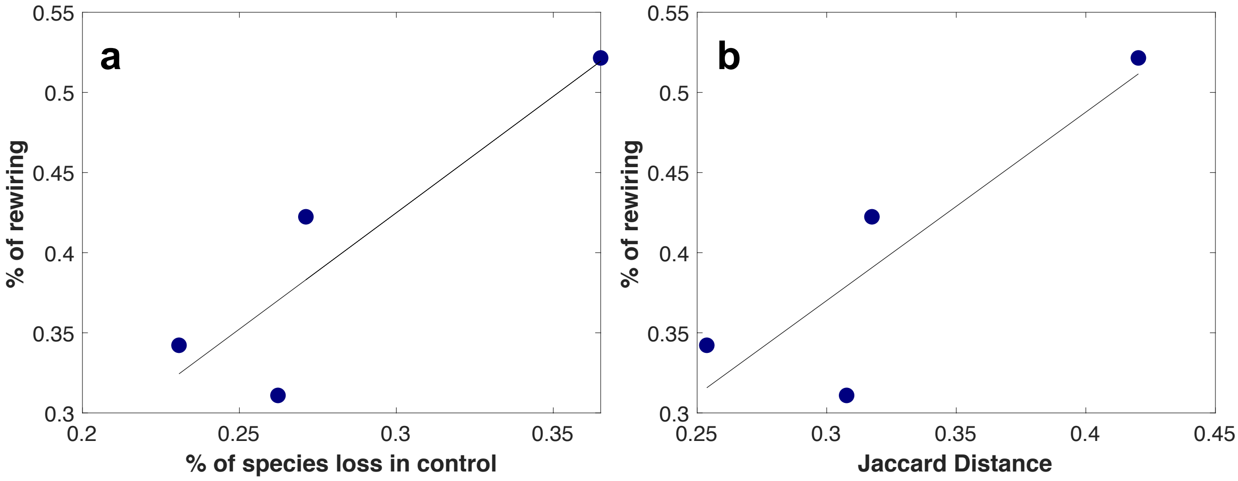

Supplementary Figure 9. The level of rewiring increases with biodiversity loss. a The proportion of rewiring found in a drought web directly increases with the amount of species loss in the corresponding control web (Linear regression: $F_{1,3}$ = 7.87, P = 0.107, r^2 = 0.80; y = -0.0108 + 1.4524x). **b** The greater the difference in the species composition between the control and drought web pairs, the higher the level of rewiring found in the drought web (Linear regression: $F_{1,3}$ = 6.21, P = 0.130, r^2 = 0.76; y = 0.0173 + 1.1759x). Both cases show that the level of rewiring, which is the proportion of new links found in the drought web that were not in the control counterpart, increases with the extent in which food webs were perturbed, albeit not significantly due to small sample size.

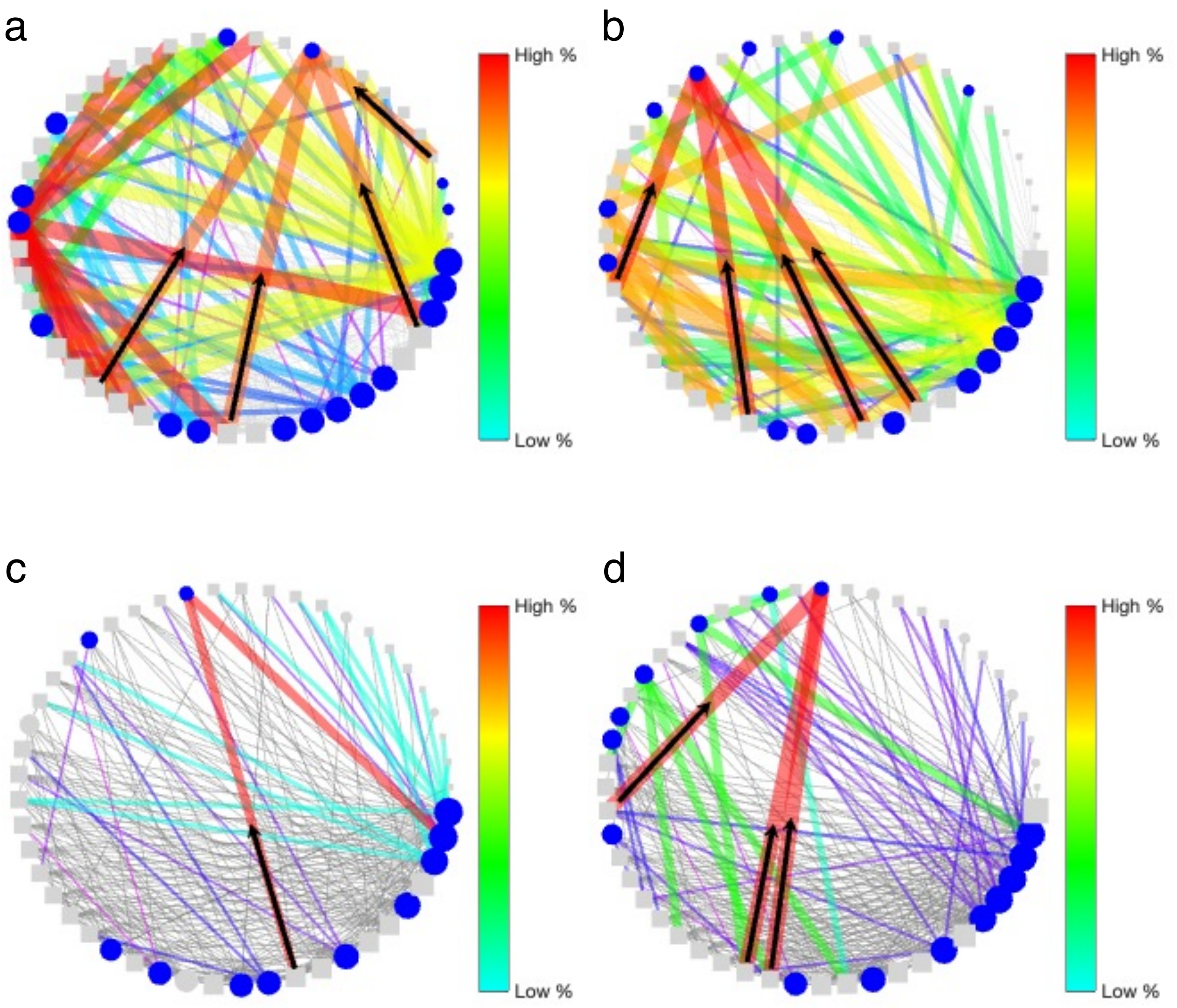

Supplementary Figure 10. Adaptability among poorly connected species governs persistence in the face of drought. a-d Drought webs 1-4, respectively. Specialist species with narrower diets were found to have rewired proportionally more than generalist species. Circles are consumers whereby those in blue have rewired, otherwise, they are in grey. Squares are resource species. All nodes are placed in ascending order of degree, indicated by node size. Links are divided into unaltered links from the control web (unweighted grey lines) and rewired links that are only found in the drought web (weighted and colour-mapped lines), with both colour and weight indicating the proportion of rewired links of a given species. An example of a low degree consumer with a high proportion of rewired links is shown in each case with the transfer of energy fluxes from new resource species indicated by dark arrows.

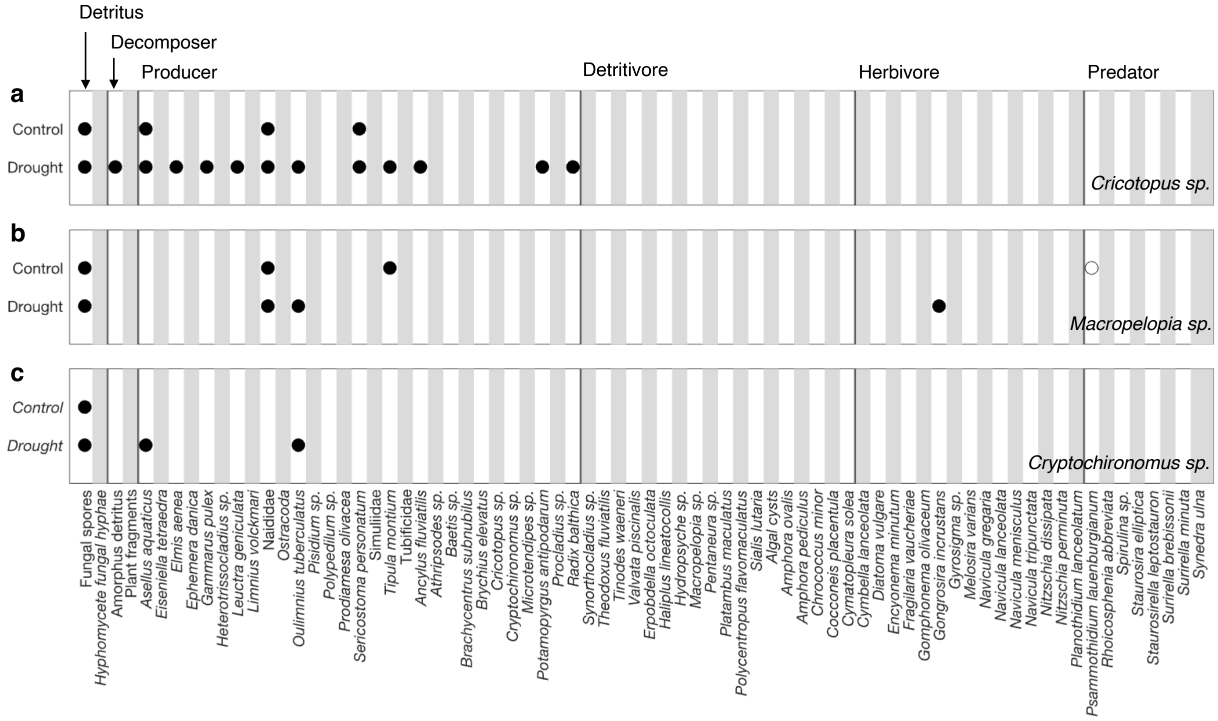

Supplementary Figure 11. Dietary shifts among the highly rewired specialists. a-c *Cricotopus sp.*, *Macropelopia sp.* and *Cryptochironomus sp.* respectively. Species on the x-axis are categorised into functional groups and then ordered by increasing order of body mass. Resources (filled circles) of a given generalists are shown in a pair of control and drought webs, with those who are extinct following drought indicated (not filled circles).
