# Supplementary material for: Network rewiring conserves the topology of drought-impaired food webs
Source: Commun Biol. 2025 Nov 24;8:1641. doi: 10.1038/s42003-025-09035-2 (PMC12644991; doi:10.1038/s42003-025-09035-2)
Supplement: Supplementary file 2 — Description of Additional Supplementary Materials [file 42003_2025_9035_MOESM2_ESM.pdf]

Description of Additional Supplementary Files

File name: Supplementary Data 1

Description: Edgelists of control and drought webs. Four control webs (tabs C1-C4) and four drought counterpart webs (tabs D1-D4) whereby each interaction link is represented by a consumer and resource pair.

File name: Supplementary Data 2

Description: The source data behind the graphs in the paper.
