# Supplementary material for: Network rewiring conserves the topology of drought-impaired food webs
Source: Commun Biol. 2025 Nov 24;8:1641. doi: 10.1038/s42003-025-09035-2 (PMC12644991; doi:10.1038/s42003-025-09035-2)
Supplement: Supplementary file 5 — Supplementary Software [file 42003_2025_9035_MOESM5_ESM.zip › Code/Magna Documentation.pdf]

MAGNA++: Maximizing Accuracy in Global Network Alignment via both node and edge conservation

Milenkovic Lab

Section 1: System Requirements

MAGNA++ runs on Linux, Mac OS X, and Windows, and is available for download as a compressed file **here**. Statically compiled executable operating systems, so the aligner is ready to run immediately after downloading, though instructions are also provided to compile directly fr

The suggested network size for MAGNA++ is up to 10,000 nodes and 50,000 edges. The aligner's memory requirement increases linearly w nodes and edges. The time taken to run the aligner increases linearly with respect to the number of edges, with the running time decreasin number of cores used.

The MAGNA++ download takes up 1-2MB of disk space, though more space will be required to store networks and the resulting alignments

Installing involves simply uncompressing the downloaded file and running MAGNA++.

Section 2: Using MAGNA++

2.1: Graphical User Interface (GUI)

Start MAGNA++ by running the executable. MAGNA++ comes with a user-friendly graphical interface; below is a screenshot of the interface each input field.

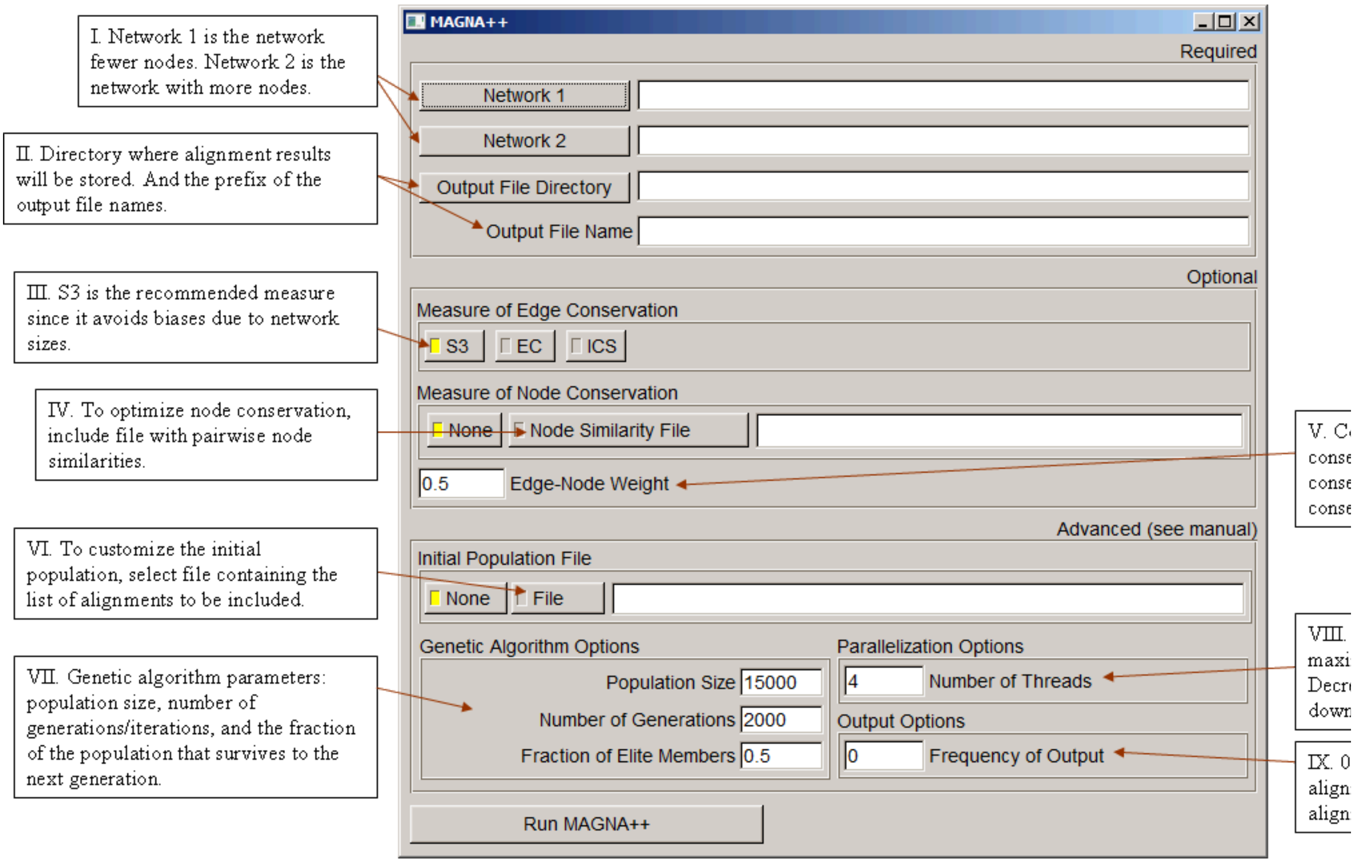

I.
Choose two networks to align. Network 1 must have fewer nodes than Network 2. MAGNA++ accepts networks in either LEDA network form edge list format (.txt) (see an **example**) or Simple Interaction Format (.sif) (see an **example**). Various real-world, synthetic, testing, and toy can be found **here**.

II.
Choose the output directory and a prefix for each output file name. For example, if "ex_" is entered, then "ex_" will appear at the beginning that MAGNA++ outputs. MAGNA++ will output files for the resulting alignments, the parameters used to create the alignments, and some st See Section 3 for a more detailed description of output files.

III.
Choose an edge-based measure to optimize. S^3 is the recommended optimization measure since it avoids biases due to network sizes.

IV.

Choose whether to optimize a node-based measure in addition to optimizing an edge-based measure. If "None" is selected, then MAGNA++ conservation (see IV). If "Node Similarity File" is selected, then choose a node similarity file. MAGNA++ can read node similarity files in tw format (see an **example**) or the CSV matrix format (see an **example**).

Supposing Network 1 has m nodes and Network 2 has n nodes. The simple matrix format should contain the size of the matrix on the first li columns delimited by spaces. The CSV matrix format should have m x n lines, the first column containing the nodes in Network 1, the secor and the third column the similarity value between the two nodes.

V.

Choose a parameter α. Using this parameter, MAGNA++ can simultaneously optimize an edge-based measure (EC, ICS, or S^3) and a nod topological or biological node similarities). In particular, given an edge-based measure S_E, a node-based measure S_N, and a parameter MAGNA++ will optimize $\alpha S_E + (1-\alpha) S_N$. When α is 1, MAGNA++ will only optimize edge conservation, and when α, MAGNA++ wil conservation.

VI.

MAGNA++ can either improve an existing set of alignments or start from scratch. Choose either "None" or "File". If "File" is selected, then M initial population file, which will define an existing set of alignments for which to improve. This file should contain a list of alignments file na which must be in the same directory as the initial population file. If "None" is selected, no initial population will be used, so MAGNA++ will s generating a random set of initial alignments.

VII.

MAGNA++ uses a genetic algorithm, for which there are three parameters:

"Population Size" is the population size of the genetic algorithm; that is, the genetic algorithm will work with this many alignments with each population is provided (see VI), then 15,000 is generally the recommended population size. If using alignments generated by other algorithr initial population, then a population size of 10,000 is recommended.

"Number of Generations" is the number of iterations or generations for which the genetic algorithm will run. If no initial population is used, t recommended number of generations. If using an alignments generated by another algorithm, then 1,000 is the recommended number of ge

"Fraction of Elite Members" is the highest-quality fraction of the population that survives to the next generation. 0.5 is always the recommen

VIII.

MAGNA++ is capable of using multiple threads to perform its computation. It parallelizes the calculation of alignment quality, which is the c MAGNA++. Calculation of alignment quality is divided among threads. MAGNA++ automatically determines the number of threads by using a are cores in the computer, though this value can be changed manually. However, using more threads than there are cores is not recommend not likely to improve. Performance will drop if too many threads are used.

IX.

Choose how often MAGNA++ outputs the best alignment in the population. If the frequency of output is k, and there are n generations, then best alignment every n/k generations. For example, if k = 1, then MAGNA++ will output the best alignment with every generation, and if k = output the best alignment of the last generation. It is recommended that k divides n.

2.2: Command Line Interface (CLI)

Instructions for running the command line interface (CLI) of MAGNA++ can be found in the README.txt file that accompanies the CLI execu

Section 3: Output and Results

Upon beginning an execution (and supposing the prefix defined in 2.II is "ex_"), MAGNA++ generates three output files by default. The final "ex_final_alignment.txt" consists of the aligned pairs of nodes calculated by MAGNA++. The alignment statistics file "ex_final_stats.txt" con similarity, and the overall score of the final alignment. The common conserved subgraph file, "ex_final_visualize.sif", contains the common alignment of the two networks. It provides an intuitive visualization of the final alignment, and can be viewed using a program such as Cyto

If the frequency of output option is set to k, then MAGNA++ outputs the details of the best alignment every n/k generations (where n is the asdefined in 2.XI). Each alignment is written to a file, containing a list of node pairs. Nodes in the left column are from Network 1 and the n Network 2. Also according to the frequency of output, MAGNA++ will append statistics of the best alignment to the file "ex_stats.txt".

The format of the output alignment file names is "ex_M_p_n_i.aln", where M is the edge conservation measure (one of EC, ICS, S3), p is th total number of generations, and i is the generation that the alignment was written. The alignment from the last generation (ex_M_p_n_n.alı produced by MAGNA++.

MAGNA++ will also create a file "ex_params.txt", which contains a summary of the initial parameters given.

Section 4: Authors, Credits, and Licensing

MAGNA++ is created by **V. Vijayan**, **V. Saraph** and **T. Milenkovic** at University of Notre Dame.

MAGNA++ is freely available for academic use only.

Disclaimer: THE SOFTWARE IS PROVIDED "AS IS", WITHOUT WARRANTY OF ANY KIND, EXPRESS OR IMPLIED, INCLUDING BUT NOT
WARRANTIES OF MERCHANTABILITY, FITNESS FOR A PARTICULAR PURPOSE AND NONINFRINGEMENT. IN NO EVENT SHALL THE AU
HOLDERS BE LIABLE FOR ANY CLAIM, DAMAGES OR OTHER LIABILITY, WHETHER IN AN ACTION OF CONTRACT, TORT OR OTHERW
OF OR IN CONNECTION WITH THE SOFTWARE OR THE USE OR OTHER DEALINGS IN THE SOFTWARE.
