# Supplementary material for: Network rewiring conserves the topology of drought-impaired food webs
Source: Commun Biol. 2025 Nov 24;8:1641. doi: 10.1038/s42003-025-09035-2 (PMC12644991; doi:10.1038/s42003-025-09035-2)
Supplement: Supplementary file 6 — Reporting Summary [file 42003_2025_9035_MOESM6_ESM.pdf]

nature portfolio

Corresponding author(s): Athen Ma

Last updated by author(s): Sep 16, 2025

Reporting Summary

Nature Portfolio wishes to improve the reproducibility of the work that we publish. This form provides structure for consistency and transparency in reporting. For further information on Nature Portfolio policies, see our Editorial Policies and the Editorial Policy Checklist.

Statistics

For all statistical analyses, confirm that the following items are present in the figure legend, table legend, main text, or Methods section.

n/a	Confirmed	
☐	☒	The exact sample size (*n*) for each experimental group/condition, given as a discrete number and unit of measurement
☐	☒	A statement on whether measurements were taken from distinct samples or whether the same sample was measured repeatedly
☐	☒	The statistical test(s) used AND whether they are one- or two-sided<br>*Only common tests should be described solely by name; describe more complex techniques in the Methods section.*
☐	☒	A description of all covariates tested
☐	☒	A description of any assumptions or corrections, such as tests of normality and adjustment for multiple comparisons
☐	☒	A full description of the statistical parameters including central tendency (e.g. means) or other basic estimates (e.g. regression coefficient) AND variation (e.g. standard deviation) or associated estimates of uncertainty (e.g. confidence intervals)
☐	☒	For null hypothesis testing, the test statistic (e.g. *F*, *t*, *r*) with confidence intervals, effect sizes, degrees of freedom and *P* value noted<br>*Give P values as exact values whenever suitable.*
☒	☐	For Bayesian analysis, information on the choice of priors and Markov chain Monte Carlo settings
☒	☐	For hierarchical and complex designs, identification of the appropriate level for tests and full reporting of outcomes
☒	☐	Estimates of effect sizes (e.g. Cohen's *d*, Pearson's *r*), indicating how they were calculated

Our web collection on statistics for biologists contains articles on many of the points above.

Software and code

Policy information about availability of computer code

Data collection	Data were collected from an outdoor stream mesocosm experiment in which benthic communities subjected to a drought treatment (a six-day drying event conducted monthly for two years) were compared with those from undisturbed controls. There were four replicates of each treatment sampled at the end of the experiment, resulting in eight food webs in total. The experimental design simulated periodic drying events occurring during a supra-seasonal drought, and details of the experimental design and methods used to build the food webs are published in Nature Climate Change 3, 223–227 (2013).
Data analysis	The data analysis conducted in this paper was done using open source tools and published methods, as described in the text.

For manuscripts utilizing custom algorithms or software that are central to the research but not yet described in published literature, software must be made available to editors and reviewers. We strongly encourage code deposition in a community repository (e.g. GitHub). See the Nature Portfolio guidelines for submitting code & software for further information.

Data

Policy information about availability of data

All manuscripts must include a data availability statement. This statement should provide the following information, where applicable:

- Accession codes, unique identifiers, or web links for publicly available datasets
- A description of any restrictions on data availability
- For clinical datasets or third party data, please ensure that the statement adheres to our policy

Data are made available as part of Supplementary Information (Supplemnentary Data 1 and 2).

Research involving human participants, their data, or biological material

Policy information about studies with human participants or human data. See also policy information about sex, gender (identity/presentation), and sexual orientation and race, ethnicity and racism.

Reporting on sex and gender	Sex and gender were not collected as part of the study
Reporting on race, ethnicity, or other socially relevant groupings	Race, ethnicity or other socially relevant groupings were not collected as part of the study
Population characteristics	This information was not collected as part of the study
Recruitment	The study did not recruit any participants
Ethics oversight	The study did not require any ethics approval

Note that full information on the approval of the study protocol must also be provided in the manuscript.

Field-specific reporting

Please select the one below that is the best fit for your research. If you are not sure, read the appropriate sections before making your selection.

☐ Life sciences ☐ Behavioural & social sciences ☒ Ecological, evolutionary & environmental sciences

For a reference copy of the document with all sections, see nature.com/documents/nr-reporting-summary-flat.pdf

Ecological, evolutionary & environmental sciences study design

All studies must disclose on these points even when the disclosure is negative.

Study description	A drought experiment was conducted in four blocks of two linear stream mesocosms (width 0.33 m, length 12 m, depth 0.30 m) sited outdoors adjacent to, and fed by, a chalk stream at the Freshwater Biological Association River Laboratory in Dorset, U.K. (see Nature Climate Change 3, 223–227 (2013) for full details). Treatments were continuous flow (control, n=4 flumes) and drought (intermittent flow, n=4 flumes).
Research sample	At the end of the experiment (after two years), the entire macroinvertebrate assemblage in each mesocosm was collected using a hand net, with samples using subsequently to construct food webs by direct observation (microscopy) of feeding links in 3,643 individuals in total across the eight food webs
Sampling strategy	At the end of the 2-year mesocosm experiment we collected all of the organisms in each flume and thus our food webs respresent the pattern of interactions occurring in each flume section at the time of sampling.
Data collection	At the end of the experiment, all invertebrates were collected and identified before gut content analysis via microscopy. All individuals and their gut contents were identified to genus or species level, where possible. The resultant eight food webs are among the most highly resolved so far, comprising 783 pairwise trophic interactions and 74 trophic elements in aggregate.
Timing and spatial scale	Over 24 months (March 2000–February 2002)
Data exclusions	No data have been excluded in the food web construction.
Reproducibility	Results on network alignment were averaged over 30 runs.
Randomization	In the mesocosm experiment, each spatial block (n=4) of flumes contained both a control and drought treatment flume, with position within block allocated randomly.

Blinding	This was not applicable to this study, as we were not conducting tests on multiple groups

Did the study involve field work? ☒ Yes ☐ No

Field work, collection and transport

Field conditions	The study was undertaken outdoors in a series of stream mesocosms (linear flumes) based outside at the Freshwater Biological Association River Laboratory. The experiment ran for 2 years, from March 2000–February 2002, and thus the ambient field conditions varied with season. We did not collect local weather or climate parameters however.
Location	Stream mesocosms sited outdoors adjacent to, and fed by, a chalk stream at the Freshwater Biological Association River Laboratory, East Stoke, Dorset, UK (50◦ 40' 48" N, 2◦ 11' 06" W), details can be found in Global Change Biol. 17, 2288–2297 (2011) and Oecologia 155, 809–819 (2008).
Access & import/export	Samples were collected from experimental habitats housed within the grounds of a research facility (FBA River Laboratory, Dorset, UK), with the full permission of the FBA director and staff. Macroinvertebrates are collected routinely as part of biomonitoring / research programmes across the UK and no special permission is required to collect them.
Disturbance	The gravel substratum habitat with the stream mesocosms was disturbed when the samples were collected. Downstream effects (e.g. sediment deposition from outflowing water) of this were limited as water flowed through a sediment trap before being discharged to the local feeder stream. This disturbance was within an experimental system, which itself enabled researchers to avoid disturbance directly to nearby rivers and streams that might otherwise have served as the primary location for this work.

Reporting for specific materials, systems and methods

We require information from authors about some types of materials, experimental systems and methods used in many studies. Here, indicate whether each material, system or method listed is relevant to your study. If you are not sure if a list item applies to your research, read the appropriate section before selecting a response.

Materials & experimental systems

n/a	Involved in the study	
☒	☐	Antibodies
☒	☐	Eukaryotic cell lines
☒	☐	Palaeontology and archaeology
☐	☒	Animals and other organisms
☒	☐	Clinical data
☒	☐	Dual use research of concern
☒	☐	Plants

Methods

n/a	Involved in the study	
☒	☐	ChIP-seq
☒	☐	Flow cytometry
☒	☐	MRI-based neuroimaging

Animals and other research organisms

Policy information about studies involving animals; ARRIVE guidelines recommended for reporting animal research, and Sex and Gender in Research

Laboratory animals	No laboratory animals were used.
Wild animals	Outdoor stream mesocosms were colonized naturally by benthic macroinertebrates (typical chalk stream fauna) from a feeder stream based at the FBA River Laboratory on the River Frome in Dorset, U.K.. They were subjected to either continuous flow, or intermittent drying, and the communities remaining in the flumes after two years of the treatments were kick sampled from the substratum and preserved immediately in formaldehyde.
Reporting on sex	Sex is not a relevant factor in this work and no data on sex were collected.
Field-collected samples	The study did not involve the work with field collected animals in a laboratory.
Ethics oversight	Ethics oversight was provided by the University of Birmingham with advice from the Freshwater Biological Association and the Centre for Ecology and Hydrology.

Note that full information on the approval of the study protocol must also be provided in the manuscript.

Plants

Seed stocks	No seed stocks were used
Novel plant genotypes	Not applicable
Authentication	Not applicable
